# Nicotinamide riboside attenuates age-associated metabolic and functional changes in hematopoietic stem cells

**DOI:** 10.1038/s41467-021-22863-0

**Published:** 2021-05-11

**Authors:** Xuan Sun, Benjamin Cao, Marina Naval-Sanchez, Tony Pham, Yu Bo Yang Sun, Brenda Williams, Shen Y. Heazlewood, Nikita Deshpande, Jinhua Li, Felix Kraus, James Rae, Quan Nguyen, Hamed Yari, Jan Schröder, Chad K. Heazlewood, Madeline Fulton, Jessica Hatwell-Humble, Kaustav Das Gupta, Ronan Kapetanovic, Xiaoli Chen, Matthew J. Sweet, Robert G. Parton, Michael T. Ryan, Jose M. Polo, Christian M. Nefzger, Susan K. Nilsson

**Affiliations:** 1 Biomedical Manufacturing Commonwealth Scientific and Industrial Research Organisation (CSIRO), Melbourne, VIC Australia; 2 Australian Regenerative Medicine Institute, Monash University, Melbourne, VIC Australia; 3 Institute for Molecular Bioscience, The University of Queensland, Brisbane, QLD Australia; 4 Monash Biomedicine Discovery Institute, Melbourne, VIC Australia; 5 Department of Anatomy and Developmental Biology, Monash University, Melbourne, VIC Australia; 6 Department of Biochemistry and Molecular Biology, Monash University, Melbourne, VIC Australia; 7 IMB Centre for Inflammation and Disease Research, The University of Queensland, St. Lucia, QLD Australia; 8 Australian Infectious Diseases Research Centre, The University of Queensland, St. Lucia, QLD Australia; 9 Centre for Microscopy and Microanalysis, The University of Queensland, St. Lucia, QLD Australia

**Keywords:** Stem cells, Haematopoietic stem cells

## Abstract

With age, hematopoietic stem cells (HSC) undergo changes in function, including reduced regenerative potential and loss of quiescence, which is accompanied by a significant expansion of the stem cell pool that can lead to haematological disorders. Elevated metabolic activity has been implicated in driving the HSC ageing phenotype. Here we show that nicotinamide riboside (NR), a form of vitamin B3, restores youthful metabolic capacity by modifying mitochondrial function in multiple ways including reduced expression of nuclear encoded metabolic pathway genes, damping of mitochondrial stress and a decrease in mitochondrial mass and network-size. Metabolic restoration is dependent on continuous NR supplementation and accompanied by a shift of the aged transcriptome towards the young HSC state, more youthful bone marrow cellular composition and an improved regenerative capacity in a transplant setting. Consequently, NR administration could support healthy ageing by re-establishing a more youthful hematopoietic system.

## Abstract (web summary)

Aged hematopoietic stem cells (HSC) are characterised by reduced regenerative potential and a loss of quiescence. Here, the authors show nicotinamide riboside treatment shrinks the age-enlarged stem cell pool and shifts aged HSC functionally, metabolically and molecularly towards the young state.

## Introduction

Ageing involves progressive deterioration of cellular function and is one of the greatest risk factors for developing a large cohort of debilitating diseases, including disorders of the hematopoietic system^1^. Hematopoietic stem cells (HSC) are responsible for the lifelong production of all circulating blood cells. With age, HSC undergo changes in function and phenotype, including compromised regenerative potential, loss of quiescence and increased metabolic activity, which is accompanied by a significant expansion of the HSC pool and differentiation skewed towards the myeloid lineage^1–3^. Consequently, HSC ageing is associated with immune senescence and can also lead to the development of haematological disorders such as anaemia and blood cancer^1,4,5^. In addition, aged HSC (donors > 45 years) have a reduced capacity to reconstitute hematopoiesis in clinical transplants, exacerbating the shortage of bone marrow (BM) donors^6,7^. Hence, agents that reverse or mitigate HSC ageing would not only have a tremendous socio-economic impact but also enable healthy ageing.

Although the molecular mechanisms underlying HSC ageing are actively debated, emerging evidence suggests changes in HSC metabolism directly contributes to the functional defects associated with ageing^8–10^. Under young homoeostatic conditions, HSC are in a metabolically inactive, primarily glycolytic and quiescent state, but can temporarily convert to a metabolically active, oxidative phosphorylation-driven and proliferative state on demand to replenish the blood system^11,12^. Upon ageing, the majority of HSC gradually leave their quiescent state to become permanently metabolically more active^10,13^. As such elevated levels of mitochondrial stress have been linked to the age-altered metabolic HSC state^14^. Autophagy is associated with health and longevity and is critical for protecting HSC from metabolic stress. Metabolic re-wiring has been associated with reduced autophagy in aged HSC, causing an accumulation of mitochondria and an activated metabolic state, impairing HSC regenerative potential^10,15^.

Nicotinamide adenine dinucleotide (NAD) and its related co-enzymes (NADH, NADP^+^ and NADPH) are central mediators of metabolic processes. NAD itself plays an essential role in maintaining metabolic homoeostasis, functioning as a hydride group receptor by accepting electrons from other metabolites derived from different metabolic pathways, to form NADH. The electron transfer properties of NAD/NADH are critical for many cellular functions, including DNA synthesis, mitochondria respiration, energy production and metabolism^16–20^. As such, a range of metabolic stressors can disturb one or more NAD metabolites, including alcohol consumption^21^, overnutrition and type 2 diabetes^22^, circadian disruption^23^, DNA damage^24^ postpartum^25^, as well as heart failure and tissue injuries^26,27^. Furthermore, CD38, a NAD degrading enzyme, is widely expressed on cells of the immune system and upregulated by an array of inflammatory mediators^28^. Thus, it is not surprising that NAD levels decline with age in many tissues and organs^29^. Therefore, NAD precursors have recently emerged as promising therapeutic agents for age-related regenerative decline and its associated diseases. Indeed, NAD precursor administration effectively restored NAD levels in a variety of tissues of aged mice, and successfully reversed the age-related stem cell dysfunction in a number of organ systems: including skeletal muscle, brain, skin and endothelium^17,30–32^. Critically, the NAD precursor nicotinamide riboside (NR)^33^ has recently been shown to decrease the overall metabolic activity of young HSC through increased mitochondrial clearance, augmenting hematopoiesis and regenerative capacity^34^. However, the metabolic, functional and molecular impact of NR treatment on aged HSC and whether this can restore a more youthful function is an unresolved question of critical importance.

Here, we report that NR administration to aged mice attenuates the age-associated metabolic and functional changes in HSC; restoring a youthful metabolism accompanied by reduced mitochondrial network size, a transcriptional shift towards the young state, a more young-like BM cellular composition and importantly, improved regenerative capacity in a transplant setting. Metabolic correction is dependent on continuous supplementation of NR, with cessation of treatment resulting in reversion to the original ageing state. The data identifies NR administration, a form of Vitamin B3 with excellent preclinical/clinical safety profiles^33,35–37^, as an avenue to re-establish a more youthful hematopoietic system to ultimately support healthy ageing.

## Results

### NR treatment improves the cellular composition of the aged bone marrow

**Fig. 1:**
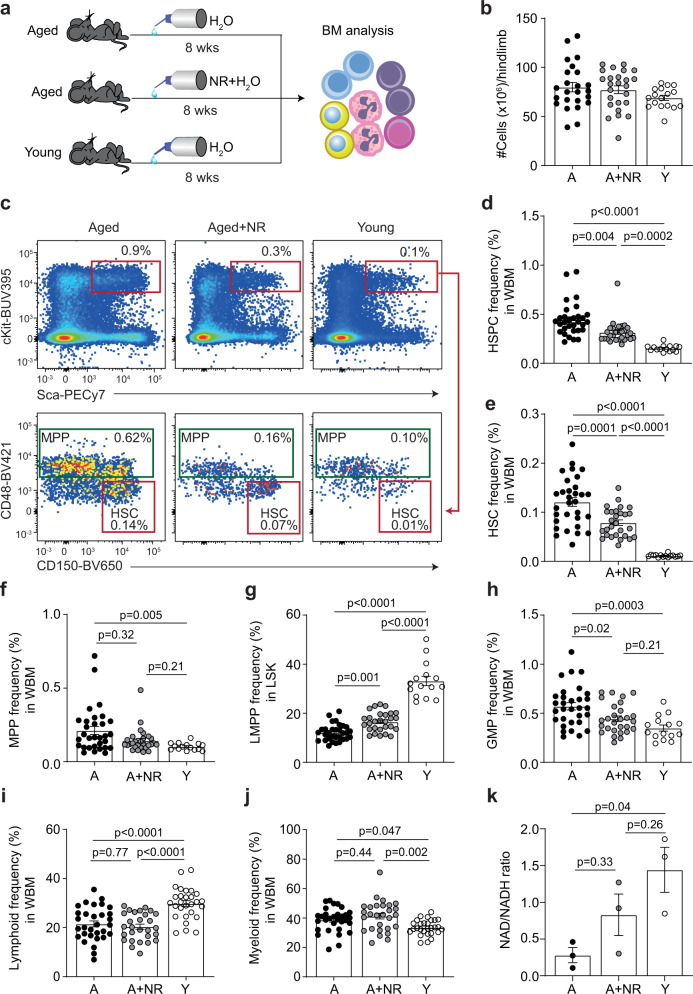
NR treatment improves the cellular composition of aged BM. **a** Schematic of the experimental setup. **b** Hind limb BM cellularity in aged (*n* = 24, biological replicates), NR-treated aged (*n* = 25, biological replicates) and young (*n* = 17, biological replicates) mice. **c** Representative flow cytometric dot plots for HSPC (Lin^−^Sca-1^+^cKit^+^), HSC (Lin^−^Sca-1^+^cKit^+^CD150^+^CD48^−^) and MPP (Lin^−^Sca-1^+^cKit^+^CD48^+^) in the BM of aged, NR-treated aged and young mice. **d**, **e** Incidence of **d** HSPC and **e** HSC in aged (*n* = 31, biological replicates), NR-treated aged (*n* = 28, biological replicates) and young (*n* = 15, biological replicates) mice. **f**–**h** Incidence of **f** MPP, **g** LMPP (Lin^−^Sca-1^+^cKit^+^CD34^+^Flt3^+^) and **h** GMP (Lin^−^CD127^−^Sca-1^−^cKit^+^CD34^+^CD16/32^+^). **i** lymphoid cells (CD3^+^, B220^+^) and **j** myeloid cells (Gr1/Mac-1^+^) in BM from aged (*n* = 31, biological replicates), NR-treated aged (*n* = 28, biological replicates) and young (*n* = 15, biological replicates) mice. Data are mean ± s.e.m. and is a pool across 8 separate collection days. Statistical analyses were performed using Kruskal–Wallis test, *p* = 0.16 (overall for **b**), *p* < 0.0001 (overall for **d**), *p* = 0.006 (overall for **f**) with individual groups compared using Dunn’s multiple comparisons test, *p*-values indicated for **b**, **d**, **f** and ordinary one-way ANOVA, *p* < 0.0001 (overall for **e**), *p* < 0.0001 (overall for **g**), *p* = 0.0003 (overall for **h**), *p* < 0.0001 (overall for **i**), *p* = 0.002 (overall for **j**) with individual groups compared using Tukey’s multiple comparisons test, *p*-values indicated for **e**, **g**, **h**, **i**, **j**. **k** Quantification of relative NAD levels in BM HSPC from aged (*n* = 3, biological replicates), NR-treated aged (*n* = 3, biological replicates) and young (*n* = 3, biological replicates) mice, represented as the ratio of NAD/NADH. NADH levels are unchanged in aged, NR-treated aged and young mice as shown in Supplementary Fig. 2l. Data are mean ± s.e.m. and statistical analysis was performed using Ordinary one-way ANOVA, *p* = 0.04 (overall for **k**) with individual groups compared using Tukey’s multiple comparisons test, *p*-values indicated. NR nicotinamide riboside, BM bone marrow, WBM total bone marrow, HSPC hematopoietic stem and progenitor cells, HSC hematopoietic stem cells, MPP multipotent progenitors, GMP granulocyte–macrophage progenitors, LMPP Lymphoid-primed multipotent progenitors, Y young, A aged, A + NR NR-treated aged animal. Source data are provided as a Source Data File.

Previous studies have utilised NR at doses of 400–500 mg/kg/day for up to 8 weeks to improve the function of aged muscle, neural and intestinal stem cells^32,38^. To align with these studies, we used an 8-week treatment of 400 mg/kg/day of NR (Fig. 1a). As expected, the average number of BM cells was not altered as mice aged^39^, nor following NR treatment (Fig. 1b). However, aged BM (20–24 months of age) displayed the characteristic altered cellular composition compared to young mice (2–3 months, Fig. 1c–j and Supplementary Fig. 1a–g), confirming previous studies^40–42^. These changes included age-enlarged hematopoietic progenitor (HSPC, Fig. 1c, d and Supplementary Fig. 1a) and stem cell pool (HSC, Fig. 1c, e and Supplementary Fig. 1b). Importantly, NR treatment of aged animals leads to a significant decrease in the age-related expansion of the stem and progenitor pool (Fig. 1c–e and Supplementary Fig. 1a, b).

In addition, the more committed progenitor populations such as multi-potent progenitors (MPP), lymphoid-primed multipotent progenitors (LMPP) and granulocyte/macrophage progenitors (GMP) were also significantly altered with age (Fig. 1f–h and Supplementary Fig. 1c–g), resulting in the expected myeloid lineage bias (Fig. 1i, j). Interestingly, NR treatment partially corrected the age-associated skewing of myeloid and lymphoid lineage cells at the progenitor level, with the incidence of LMPP significantly increased (Fig. 1g and Supplementary Fig. 1d–f) and the frequency of GMP significantly reduced (Fig. 1h and Supplementary Fig. 1d, e, g). However, the overall percentage of differentiated lymphoid and myeloid lineage cells was not significantly affected by NR treatment (Fig. 1i, j). This suggests that while NR had a normalising effect on the frequency of myeloid and lymphoid progenitors, treatment did not correct the age-disturbed abundance of differentiated lymphoid and myeloid cells.

In contrast to the pronounced effects of NR administration on the cellular composition of the aged BM compartment, 8 weeks of NR administration in young mice (treatment from 4 weeks of life) had no significant effects on the cellular BM composition compared to untreated animals (Supplementary Fig. 2a–k).

As reported for other tissues, including liver and skeletal muscle^17,19,32^, HSPC in aged mice had significantly reduced NAD compared to young mice (Fig. 1k and Supplementary Fig. 2l). Oral administration of NR to aged mice (20–22 months old) for 8 weeks boosted NAD levels to an intermediate state between young and aged HSPC, although the rescue from aged levels of NAD with treatment did not reach significance (Fig. 1k and Supplementary Fig. 2l). Together, the data demonstrate NR-mediated NAD replenishment partially attenuates the age-related changes in the cellular composition of the BM, most notably by decreasing the size of the age-expanded stem and progenitor cell pools.

### NR treatment shifts the aged HSC transcriptome towards a more youthful state

**Fig. 2:**
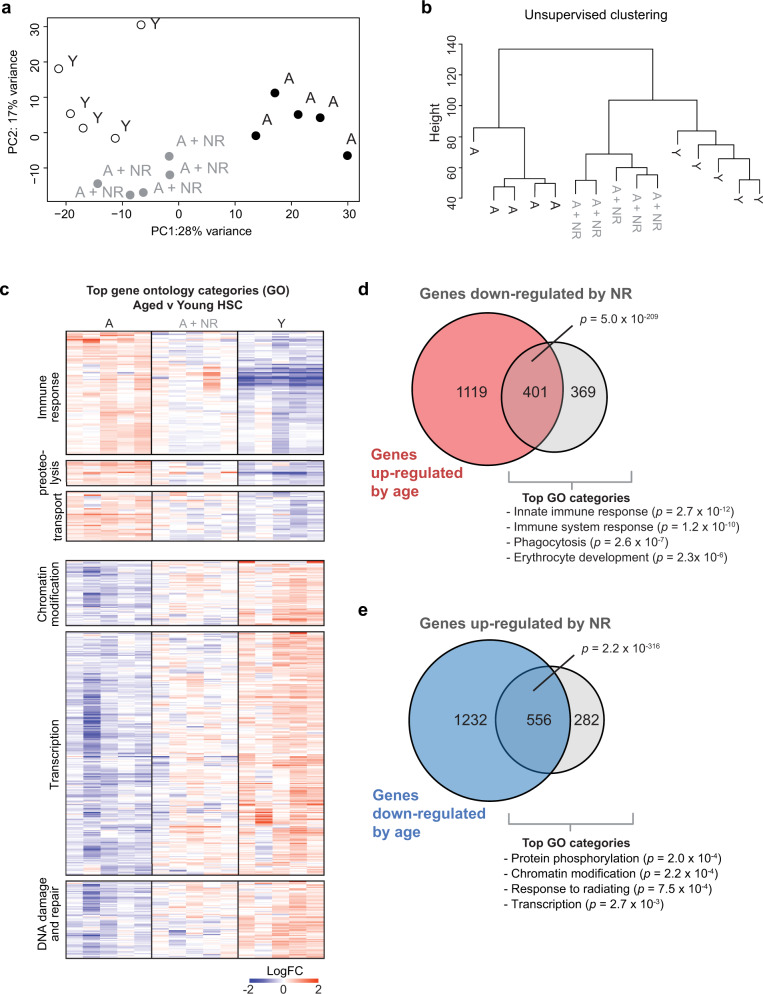
NR treatment shifts the aged HSC transcriptome towards a more youthful state. **a** Principal component analysis of RNA sequencing dataset for HSC from aged (*n* = 5, biological replicates), NR-treated aged (*n* = 5, biological replicates) and young (*n* = 5, biological replicates) mice. **b** Unsupervised clustering of the HSC RNAseq samples using all genes. **c** Heatmap representation of genes associated with key gene ontology categories for differentially expressed genes between young and aged HSC (false discovery rate < 0.05); the upper block is for age-upregulated genes, the lower block for age downregulated genes. **d** Venn diagram visualising overlap between age-upregulated genes (Y vs A, false discovery rate < 0.05) and genes downregulated by NR exposure (A vs A = NR, false discovery rate < 0.05) with associated gene ontology categories. **e** Venn diagram visualising overlap between age downregulated genes (Y vs A, false discovery rate < 0.05) and genes upregulated by NR exposure (A vs A = NR, false discovery rate < 0.05) with the associated gene ontology categories. Statistical analysis was performed using EdgeR (**c**) DAVID (**c**–**e**), or hypergeometric test with the R function phypher using lower.tail = FALSE with no multiple test correction (**d**, **e**). NR nicotinamide riboside, HSC hematopoietic stem cells, *p* = *p*-value, Y young, A aged, A + NR NR-treated aged animal.

As HSC give rise to all hematopoietic cell types, we performed RNA sequencing (RNAseq) on HSC from young, aged and NR-treated aged animals to assess if a more youthful cellular BM composition was accompanied by molecular changes. A principal component analysis (PCA) (Fig. 2a) confirmed aged and young HSC cluster discretely from each other and agrees with previous reports^43–45^. 3388 genes were differentially expressed between young and aged HSC (Supplementary Data 1). Comparison of the age-regulated genes from a previously published study using comparable age groups and a similar HSC isolation strategy demonstrated significant overlap (Supplementary Fig. 3a)^45^, indicating both studies detect similar aspects of an aged HSC transcriptional signature.

HSC from NR-treated aged animals clustered in an intermediate position between the aged and young HSC, suggesting NR treatment shifted the transcriptome of aged HSC towards a more youthful state (Fig. 2a). Indeed, unsupervised hierarchical clustering positioned HSC from NR-treated aged animals closer to young HSC than to aged HSC (Fig. 2b).

The top-ranked gene ontology categories for age-upregulated genes (Y vs A, FDR < 0.05, Supplementary Data 1) were associated with immune response, proteolysis and transport, while age downregulated genes (Y vs A, FDR < 0.05, Supplementary Data 1) were associated with chromatin modification, transcription and DNA damage and repair (Fig. 2c). The transcriptional signature of aged HSC from NR-treated mice for these gene sets was intermediate between young and aged (Fig. 2c). As such, the number of differentially expressed genes between young and aged HSC dropped considerably following NR treatment (3388 genes for young vs aged controls; 1036 gene for young vs aged + NR; Supplementary Fig. 3b, c, Supplementary Data 1 and 2). Accordingly, there was a highly significant overlap between age-upregulated genes and genes downregulated in response to NR treatment (Fig. 2d, e and Supplementary Data 1, 3). Similarly, there was a highly significant overlap between genes downregulated by age and genes upregulated in response to NR treatment (Fig. 2d, e and Supplementary Data 1, 3). Together, these data suggest NR treatment is reversing the transcriptional ageing response in HSC. Specifically, genes downregulated in response to NR treatment were associated with immune response, phagocytosis and erythrocyte development (Fig. 2d), while genes upregulated by NR treatment were associated with protein phosphorylation, chromatin modification, response to radiation and transcription (Fig. 2e).

Interestingly genes that remained differentially expressed following treatment were associated with immune response-related processes and cell proliferation (Supplementary Fig. 3c), suggesting that while NR treatment normalised a large part of the age-altered transcriptional signature (Fig. 2a–e and Supplementary Fig. 3b, c), only partial correction of immune responses is observed (Fig. 2c, d) since the major remaining differences between young and NR-treated aged HSC are still associated with this GO category (Supplementary Fig. 3c). Similarly, the RNAseq data (Supplementary Fig. 3c) suggests NR treatment does not entirely overcome the reported differences in cell cycle between young and aged HSC^46,47^. Previous reports demonstrated that aged HSC have reduced cell cycle activity compared to young HSC^46^. In support of this, cell cycle analysis using Ki67 showed young HSC and HSPC had a significantly higher proportion of actively cycling cells compared to aged HSC and HSPC. NR treatment significantly increased the proportion of cycling HSPC to levels comparable to young HSPC (Supplementary Fig. 4a, b). In contrast, NR treatment only shifted the proportion of cycling HSC to an intermediate state in between young and aged cells (Supplementary Fig. 4a, c), which is consistent with our transcriptional data (Supplementary Fig. 3c). Together the transcriptional characterisation revealed NR treatment of aged animals shifted the expression levels of a subset of age-deregulated HSC genes towards a youthful state.

### NR treatment restores youthful metabolism in aged HSC

**Fig. 3:**
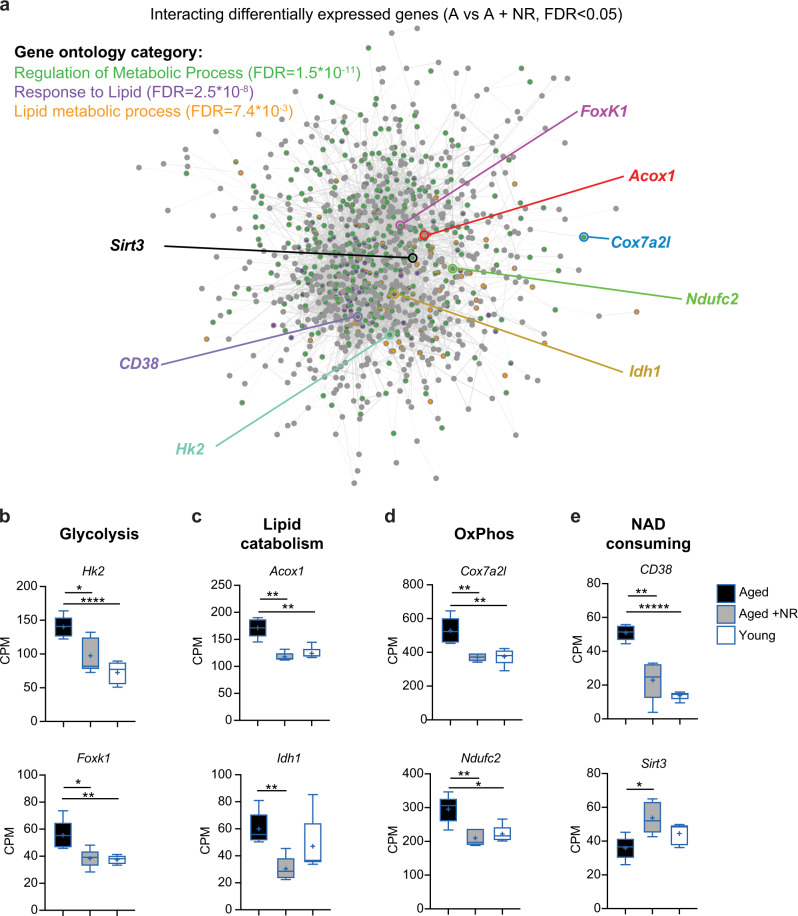
NR treatment modulates the expression of metabolic genes. **a** String-db protein–protein interaction map for differentially expressed genes between aged and NR-treated aged HSC (false discovery rate < 0.05); genes associated with gene ontology categories related to metabolic regulation are indicated by coloured nodes. **b**–**e** Expression levels of key genes associated with glycolysis, lipid catabolism, oxidative phosphorylation and NAD consumption in aged HSC (*n* = 5, biological replicates), NR-treated aged (*n* = 5, biological replicates) and young HSC (*n* = 5, biological replicates). Statistical analysis was performed using String-db (**a**) and EdgeR (false discovery rates between two groups were determined considering the third group as background, **b**–**e**). Data are expressed as box-and-whisker plots. Box indicates 25th to 75th percentile, line indicates the median and whiskers indicate min to max values. *FDR < 0.05, **FDR < 0.01, ***FDR < 0.001, ****FDR < 0.0001, *****FDR < 0.00001. Only statistically significant comparisons are indicated. Exact FDR values comparing individual groups associated with **b**–**e** are presented in Supplementary Fig. 3d. NR nicotinamide riboside, HSC hematopoietic stem cells, OxPhos oxidative phosphorylation, CPM TMM-corrected counts per million sequencing reads, Y young, A aged, A + NR NR-treated aged animal. Source data are provided as a Source Data File.

Given the crucial role of NAD in cell metabolism and the results of our molecular characterisation, we next investigated how NR repletion affects HSC metabolism. A String protein–protein interaction map^48^ for NR regulated genes (A vs A + NR, FDR < 0.05, logFC > 2) revealed an interconnected network with a high number of nodes significantly associated with regulation of metabolism (Fig. 3a). Mirroring the results of Sun et al.^45^, genes associated with primary metabolism were age-upregulated in our HSC RNAseq dataset, including *Hk2* (catalyses the first step of glycolysis^49^, Fig. 3b), *Acox1* (first enzyme of the fatty acid beta-oxidation pathway^50^, Fig. 3c) and *Cox7a2l* (mitochondrial Complex III Binding Protein that stabilises the III2 + IV Supercomplex^51^, Fig. 3d). In addition, age deregulation of NAD consuming factors was detected in HSC (Fig. 3e), including *CD38* as previously reported^45^. NR treatment significantly countered age-upregulation of *Hk2*, *Acox1*, *Cox7a2l* and *CD38* to expression levels not significantly different to those in young HSC (Fig. 3b–e). In addition, NR treatment downregulated other genes associated with metabolism including *FoxK1* (transcriptional regulator of glucose metabolism^52^, Fig. 3b), *Idh1* (isocitrate dehydrogenase^53^, Fig. 3c), and *Ndufc2* (accessory subunit of the mitochondrial membrane respiratory chain NADH dehydrogenase^54^, Fig. 3d), and upregulated others such as the mitochondrial NAD consuming deacetylase sirtuin 3 (*Sirt3*, a sirtuin family member associated with promoting mitophagy^55,56^, Fig. 3e). Overall, this suggests NR treatment promotes a state of reduced metabolic potential at the transcriptional level.

**Fig. 4:**
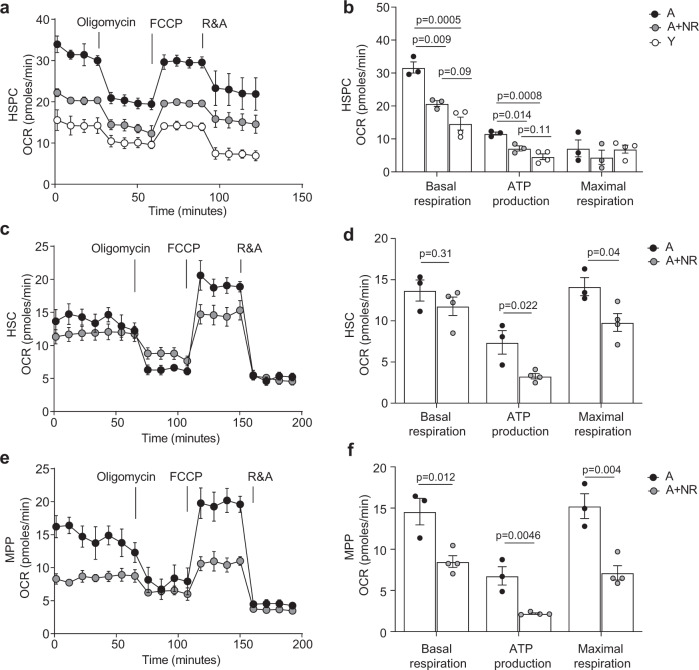
NR treatment restores youthful metabolism in aged HSC. **a** Oxygen consumption rate (OCR) in freshly isolated HSPC from aged (*n* = 3, biological replicates), NR-treated aged (*n* = 3, biological replicates) and young (*n* = 4, biological replicates) mice were measured under basal conditions followed by subsequent addition of the ATPase inhibitor oligomycin, the uncoupling reagent FCCP and the inhibitors of the electron transport chain rotenone/antimycin A (R&A), as indicated. **b** Basal respiration, ATP production and maximal respiration levels were calculated for HSPC (and throughout the manuscript) as indicated in Supplementary Fig. 5a. **c** Quantification of OCR in freshly isolated HSC and **d** determination of basal respiration, ATP production and maximal respiration capacity in aged (*n* = 3, biological replicates) and NR-treated aged mice (*n* = 4, biological replicates). **e** Quantification of OCR in freshly isolated MPP and **f** determination of basal respiration, ATP production and maximal respiration capacity in aged (*n* = 3, biological replicates) and NR-treated aged mice (*n* = 4, biological replicates). Data are mean ± s.e.m and statistical analyses were performed using Ordinary one-way ANOVA, *p* = 0.023 (overall for HSPC basal respiration), *p* = 0.0001(overall for HSPC ATP production), *p* = 0.57 (overall for HSPC maximal respiration capacity) with individual groups compared using Tukey’s multiple comparisons test, *p*-values indicated for **b** and two-sided Student’s *t*-test, *p*-values indicated for **d**, **f**. NR nicotinamide riboside, A aged, A + NR NR-treated aged animal. Source data are provided as a Source Data File.

Considering a hallmark of murine and human aged HSC is increased energy demand via mitochondrial oxidative phosphorylation (OXPHOS) compared to young HSC, which are largely quiescent and therefore in a low metabolic state^9,10^, we investigated this pathway in more detail. Seahorse metabolic flux analysis (Supplementary Fig. 5a) of mitochondrial metabolism in stem and progenitors (HSPC) demonstrated a significantly higher capacity for basal respiration and increased ATP production (Fig. 4a, b). Strikingly, NR administration in aged mice significantly reduced the capacity for basal respiration and ATP production in aged stem and progenitors (HSPC) to levels indistinguishable from young (Fig. 4a, b). Conversely, NR administration in young mice only mildly (but significantly) reduced HSPC capacity for basal respiration and ATP production (Supplementary Fig. 5b, c), consistent with previous findings^34^.

Furthermore, analysis of the effects of NR on aged HSC and progenitor subsets (Fig. 1c) demonstrated a pronounced effect on metabolism with a significant reduction in the capacity for basal respiration, ATP production and maximum respiratory capacity when compared to their untreated aged counterparts (Fig. 4c–f). Considering HSC are extremely rare in young animals, analyses of this purified cell type could only be performed using HSC isolated from NR-treated and untreated aged animals. Collectively, the data demonstrate NR repletion reduces oxygen consumption rates in aged HSPC and HSC resulting in the lower rates of ATP production associated with a youthful state.

### NR treatment mitigates stress response and contracts mitochondrial network size

**Fig. 5:**
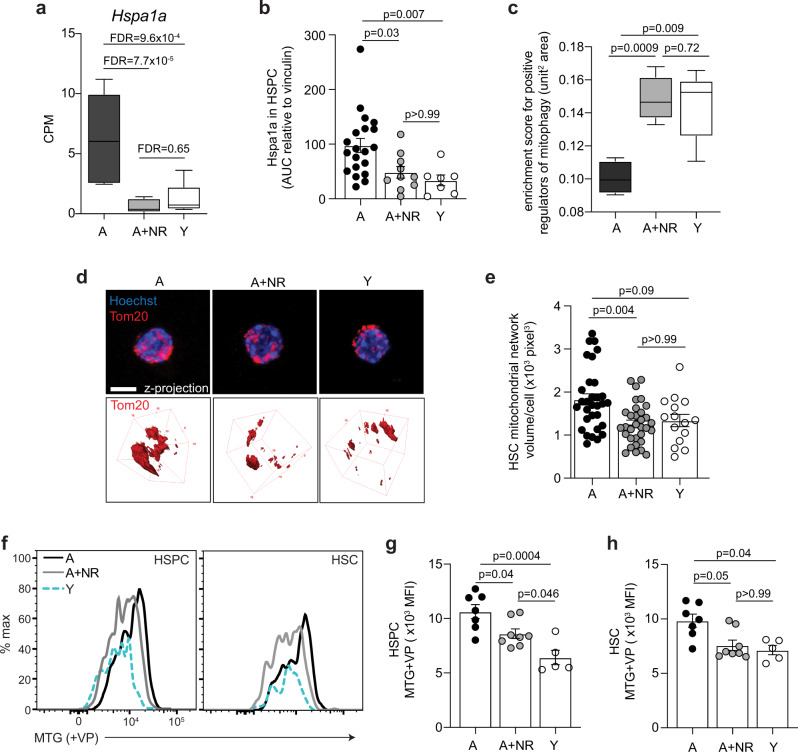
NR treatment decreases stress response and mitochondrial network size. **a** mRNA expression levels of heat shock protein *Hspa1a* in aged (*n* = 5, biological replicates), NR-treated aged (*n* = 5, biological replicates) and young HSC (*n* = 5, biological replicates). Data are expressed as box-and-whisker plots. Box indicates 25th to 75th percentile, the line indicates the median and whiskers indicate min to max values. Statistical analysis was performed using EdgeR package, false discovery rates between two groups were determined considering the third group as background. **b** Quantification of Hspa1a protein levels in aged (*n* = 20, biological replicates), NR-treated aged (*n* = 10, biological replicates) and young (*n* = 7, biological replicates) HSPC via western blot assay. Data are mean ± s.e.m. and is a pool across three separate collection days. Statistical analysis was performed using Kruskal–Wallis, *p* = 0.0019 (overall) with individual groups compared using Dunn’s multiple comparisons test, *p*-values indicated for **c**. Gene set activity analysis for positive regulators of mitophagy (*n* = 5); the *y* axis shows the score as the area under the curve and has a unit of the standardised squared unit area in the range [0,1]. Data are expressed as box-and-whisker plots and is representative of a single experiment. Box indicates 25th to 75th percentile, the line indicates the median and whiskers indicate min to max values. Statistical analysis was performed using SCENIC based pipeline, employing a two-sided Student’s *t*-test; *p*-values adjusted for multiple testing using Benjamini–Hochberg (BH) correction method are indicated. **d** Representative Tom20 immunofluorescence staining (2D and 3D volumetric) and **e** 3D volumetric quantification of total cell mitochondrial network size (μm^3^)/HSC isolated from aged (*n* = 7, biological replicates), NR-treated aged (*n* = 5, biological replicates) and young (*n* = 4, biological replicates) mice, two independent experiments with 15–20 HSC quantified from each biological sample in each experimental repetition. Scale bar = 5 μm. Data are mean ± s.e.m. and statistical analysis was performed using Kruskal–Wallis, *p* = 0.0036 (overall) with individual groups compared using Dunn’s multiple comparisons test, *p*-values indicated. Determination of relative mitochondrial mass via Mitotracker green labelling in the presence of Verapamil; **f** representative histograms for mitotracker green level in HSPC and HSC from aged, NR-treated aged and young mice, and **g** relative quantification via mean fluorescence intensity for HSPC and **h** HSC from aged (*n* = 7, biological replicates), NR-treated aged (*n* = 8, biological replicates) and young (*n* = 5, biological replicates) mice. Data are mean ± s.e.m. and is representative of two independent experiments. Statistical analyses were performed using ordinary one-way ANOVA, *p* = 0.0005 (overall) with individual groups compared using Tukey’s multiple comparisons test, *p*-values indicated for **g** and Kruskal–Wallis, *p* = 0.012 (overall) with individual groups compared using Dunn’s multiple comparisons test, *p*-values indicated for **h**. NR nicotinamide riboside, A aged, A + NR NR-treated aged animal, MTG Mitotracker green, VP verapamil, MFI mean fluorescence intensity. Source data are provided as a Source Data File.

To further expand our observation that *Sirt3* is upregulated following NR treatment, which has been implicated in reducing mitochondrial stress^14^, we investigated expression levels of heat shock protein Hspa1a, which is a potentiator of mitochondrial stress^57^. Furthermore, Hspa1a expression has been shown to promote oxygen consumption and oxidative phosphorylation in other cell systems^57–59^. Our results show age-elevated levels of *Hspa1a* at both the transcriptional and protein level (Fig. 5a, b), providing a further mechanism for age-increased metabolic activity in HSPC and HSC. Importantly transcriptional and protein levels of *Hspa1a* were significantly reduced following NR treatment (Fig. 5a, b), aligning with reduced metabolic output (Fig. 4a–d).

Mitochondria are highly versatile organelles that undergo constant fission and fusion, and this morphological robustness allows them to be organised into a highly dynamic regulatory network for cellular energy production. Since NR was demonstrated to promote mitophagy in young HSC, which was linked to improved function and reduced metabolic activity^34^, we examined whether NR had a transcriptional effect on genes associated with mitophagy in aged HSC. A rank-based area enrichment analysis for positive regulators of mitophagy demonstrated NR-treated aged HSC had significantly higher enrichment compared to aged HSC, reaching levels indistinguishable to young HSC (Fig. 5c). To confirm our transcriptional analyses, we quantified the mitochondrial network size and assessed mitochondrial mass. Crucially, NR treatment of aged mice significantly reduced the mitochondrial network size in HSC, as determined with Tom20 immunolabelling, to become not significant to that of young HSC (Fig. 5d, e and Supplementary Fig. 5d). Likewise labelling of HSPC and HSC with mitotracker green (in the presence of Verapamil to prevent dye exclusion^60,61^), demonstrated NR treatment resulted in a significant reduction of age-increased mitochondrial mass in HSPC and a reduction in HSC to levels not significantly different to those of young cells (Fig. 5f–h). To overcome image resolution limitations of the Tom20 immunolabelling (Fig. 5d) preventing a proper assessment of mitochondrial morphology, we performed electron microscopy. Analysis of young, aged and aged NR-treated HSC using electron microscopy revealed no differences in mitochondrial structure between the three groups (Supplementary Fig. 5e), suggesting metabolic changes (Fig. 4c, d) are not linked to altered mitochondrial morphology.

In summary, NR treatment decreased *Hspa1a* levels, a heat shock protein shown to increase metabolic activity in other cell systems^57–59^. Furthermore, our results indicate that NR-mediated reduction in mitochondrial network size and mass contributes to re-establishing youthful metabolic potential in aged HSPC and HSC.

### Metabolic restoration is specific to HSPC and dependent on continued NR administration

Given NR repletion significantly lowered the metabolic activity of aged HSPC to resemble the profile of young HSPC, we examined whether NR administration also lowered the metabolic potential of differentiated mature hematopoietic cell types, including macrophages, neutrophils and B cells. In contrast to stem and progenitor cells (Fig. 4a–d), no significant metabolic differences were detected between young and aged macrophages, neutrophils or B cells (Supplementary Fig. 6a–f), demonstrating metabolic dysfunction is not a general attribute shared by all aged hematopoietic cell types. Furthermore, the administration of NR to aged animals did not result in any significant changes in metabolic potential in these mature cell types (Supplementary Fig. 6a–f).

Considering NR treatment had no impact on immune cell metabolism (Supplementary Fig. 6a–f) and that the primary remaining transcriptional differences between young and aged HSC from NR-treated mice were related to immune system response (Supplementary Fig. 3c), it was anticipated that immune activation in committed immune cell populations was not impacted. Indeed, while analysis of macrophages, neutrophils and B cells for expression of the early activation marker CD69 showed a significant age-associated increase in CD69 levels on macrophages (Supplementary Fig. 6g), NR treatment was not observed to dampen age-related immune cell activation (Supplementary Fig. 6g).

**Fig. 6:**
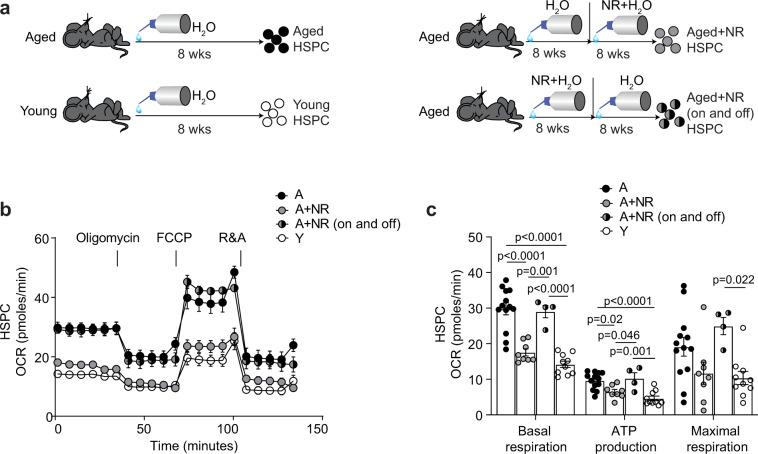
Metabolic restoration is dependent on continued administration of NR. **a** Schematic representation of the experimental setup. **b** Quantification of oxygen consumption rate (OCR) in freshly isolated HSPC from aged mice (*n* = 13, biological replicates), NR-treated aged mice (*n* = 8, biological replicates), aged mice receiving NR prior to 2 months rest (*n* = 4, biological replicates) and young mice (*n* = 10, biological replicates), as described in **a**. OCR was measured under basic conditions followed by subsequent addition of the oligomycin, FCCP and R&A, as indicated. **c** Basal respiration, ATP production and maximal respiration levels in aged (*n* = 13, biological replicates), NR-treated aged (*n* = 8, biological replicates), aged mice receiving NR prior to 2 months rest (*n* = 4, biological replicates) and young HSPC (*n* = 10, biological replicates). Data are mean ± s.e.m. and is a pool from three independent experiments. Statistical analyses were performed using ordinary one-way ANOVA, *p* < 0.0001 (overall for basal respiration), <0.0001 (overall for ATP production), *p* = 0.0084 (overall for maximal respiration capacity) with individual groups compared using Tukey’s multiple comparisons test, *p*-values indicated for **c**. Only statistically significant comparisons of individual groups are indicated. NR nicotinamide riboside, HSPC hematopoietic stem and progenitor cells, Y young, A aged, A + NR NR-treated aged animal. Source data are provided as a Source Data File.

As NAD is a co-factor for histone-modifying enzymes including sirtuins^14,62,63^ that can modify the cellular epigenome, we next investigated whether NR-mediated metabolic correction of aged stem and progenitors is stable even after cessation of NR treatment or dependent on continuous NAD precursor supplementation. We, therefore, administered NR to aged mice for 8 weeks then discontinued administration for a further 8 weeks (Fig. 6a). Strikingly, discontinuation of NR treatment completely reverted the improved metabolic phenotype (i.e. basal respiration, ATP production, maximum respiratory capacity) from aged NR-treated animals to be not significantly different from that of untreated aged stem and progenitors (Fig. 6b, c); identifying the requirement for sustained NR administration for the beneficial modulation of aged stem and progenitor metabolism.

### NR administration improves the reconstitution potential of aged HSC

Even with NR treatment improving the molecular and metabolic profiles of aged stem and progenitors, it was essential to address whether this extended to their hematopoietic function. Analysis of the potential of HSC to home to the BM post-transplant revealed a significant impairment with ageing (Supplementary Fig. 7a, b), which is consistent with previous reports^40,64^. Following NR treatment, the homing ability of aged HSC is no longer significantly different to young HSC (Supplementary Fig. 7a, b), suggesting NR supplementation shows a trend towards improvement in this age-related impairment.

**Fig. 7:**
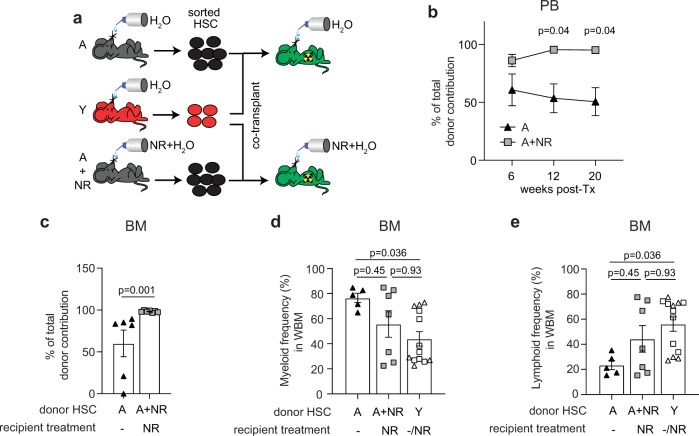
NR administration significantly improves HSC reconstitution potential of aged HSC. **a** Schematic representation of the experimental set up for the competitive transplant experiment. **b** Quantification of the percentage of aged (*n* = 6, biological replicates) and NR-treated aged (*n* = 7, biological replicates) donor cells relative to total donor-derived CD45^+^ hematopoietic cells in PB of young recipient mice at 6, 12, and 20 weeks and **c** BM at 20 weeks following transplantation. Data are mean ± s.e.m. and are representative of a single experiment. Statistical analysis was performed using two-way ANOVA with Geisser-Greenhouse correction, *p* = 0.01 (overall for treatment factor) with individual groups for each timepoint compared using Holm–Sidak multiple comparisons test, *p*-values indicated for **b** and two-sided Mann–Whitney *U*-test for **c**. **d**, **e** Quantification of the frequency of aged (*n* = 5, biological replicates), NR-treated aged (*n* = 12, biological replicates. Circle symbols refer to untreated recipients and square symbols refer to NR-treated recipients) **d** myeloid cells (Gr1/Mac-1^+^) and **e** lymphoid cells (B220^+^, CD3^+^) in BM of recipient mice 20 weeks after transplantation. For recipients where <0.01% engraftment of the relevant cell population was observed, no lineage analysis was performed. Data are mean ± s.e.m. and statistical analyses were performed using Kruskal–Wallis, *p* = 0.042 (overall) with individual groups compared using Dunn’s multiple comparisons test, p-values indicated for (**d**, **e**). Triangle symbols refer to untreated recipients and square symbols refer to NR-treated recipients. BM bone marrow, WBM total bone marrow, HSC hematopoietic stem cells, NR nicotinamide riboside, PB peripheral blood, Y young, A aged, A + NR NR-treated aged animal. Source data are provided as a Source Data File.

A more critical hallmark of aged HSC is poor BM reconstitution following transplantation. We, therefore, tested whether NR administration also enhanced the hematopoietic potential of aged HSC in a competitive transplant assay, whereby aged HSC or NR-treated aged HSC were competitively co-transplanted with young HSC (Fig. 7a). Considering that improved metabolic activity of NR-treated aged HSC was dependent on continued supplementation of the NAD precursor (Fig. 6a–c), NR was supplied to the young transplant recipients that received donor NR-treated aged HSC for the duration of the 20-week engraftment period (Fig. 7a). NR-treated aged HSC were associated with significantly improved engraftment in both peripheral blood (Fig. 7b) and BM (Fig. 7c) compared to recipients transplanted with aged HSC, demonstrating the superior regenerative potential of NR-treated aged HSC. Notably, no significant difference in myeloid and lymphoid reconstitution was observed between donor-derived NR-treated aged HSC and young HSC, demonstrating partial correction of the myeloid bias (Fig. 7d, e). However, NR treatment did not significantly alter the HSPC pool (Supplementary Fig. 8a), although a trend of decreased frequency of donor-derived HSC, MPP and GMP was evident following a transplant of NR-treated HSC compared to aged HSC (Supplementary Fig. 8b–d). No change in the frequency of LMPP was detected following a transplant of NR-treated HSC (Supplementary Fig. 8e). Importantly, NR treatment of both donor and recipients was essential for the improved hematopoietic potential of transplanted aged HSC, with untreated aged HSC transplanted into NR-treated recipients failing to give improved engraftment (Supplementary Fig. 9a–e). In addition, in vitro treatment of aged HSC with NR for 48 h was also insufficient to produce improved engraftment outcomes (Supplementary Fig. 9f–j). Likewise, transplants of NR-treated aged HSC into untreated recipients also had the equivalent hematopoietic potential to untreated aged HSC (Supplementary Fig. 10a–f).

Taken together the data demonstrate the major age-associated changes and functional deficits of HSC, in particular metabolic shifts, and compromised reconstitution potential can be restored to a more youthful state through NR-mediated NAD repletion, although the well documented age-related hematopoietic lineage bias and homing capacity are only partially corrected.

## Discussion

Stem cell ageing and its associated functional decline have long been considered an irreversible process^65^. Recent advances, however, have challenged this paradigm by highlighting the critical role of NAD homoeostasis. As such, NAD repletion is an effective therapeutic intervention for reverting the age-associated functional decline of multiple tissue-resident stem cells^17,30–32^. Furthermore, the NAD precursor NR potently induces clearance of defective mitochondria in young HSC leading to augmented regenerative potential in a transplantation setting and enhanced hematopoiesis under homoeostatic conditions^34^.

Increased metabolic output has been linked to the dysfunction of aged HSC^9^, with only a small subset of aged HSC retaining the ability of effective mitochondrial clearance to maintain a low metabolic state with high regenerative potential^10^. Therefore, enhancing mitophagy in aged HSC is a promising avenue to improve their compromised function.

The current data reveals that NR promotes a transcriptional response consistent with increased mitophagy and reduced metabolic output in aged HSC. Mitophagy promoting genes are significantly more enriched in aged HSC from NR-treated mice with the expression of *Sirt3*, implicated in regulating mitophagy^56^ and the HSC stress and regenerative response^14^, stimulated. In addition, age-elevated levels of the heat shock protein Hspa1a were returned to youthful levels. This is important as the knockdown of Hspa1a reduces oxygen consumption and ATP production in other cell systems^57–59^. Furthermore, genes associated with primary metabolic processes were downregulated by NR administration. Importantly, NAD repletion restores mitochondrial network size and mass to levels not significantly different to those of young HSC. Intriguingly this coincided with the restoration of the low metabolic activity associated with a young state. Considering the rarity of HSC and even HSPC, more direct methods such as liquid chromatography and mass spectrometry to quantify NAD levels and dissect the metabolomic impact of NR treatment on the aged stem and progenitor cells are not feasible for this cell system. Notwithstanding these limitations, our data suggest the reduced metabolic capacity of aged HSC in response to NR treatment is mediated by modifying mitochondrial function in three ways: (1) Reduced expression of metabolic pathway genes encoded in the nucleus (including *Hk2* and *Acox1*) to diminish the production of metabolic substrates; (2) Damping mitochondrial stress (i.e. downregulation of *Hspa1a* and upregulation of *Sirt3*) shown to decrease oxygen consumption and ATP production in other cell systems^57–59^; and (3) Decreased mitochondrial mass and network-size as a further contributor to reduced metabolic output.

Despite these positive effects on HSC metabolism, NR-mediated remodelling was not indefinite, with discontinuation of treatment resulting in a reversion to a highly metabolically active state. Accordingly, any potential therapeutic intervention with NR to mitigate HSC ageing will require ongoing treatment.

Functionally, under homeostatic conditions, NR treatment led to a significant reduction of the age-enlarged stem and progenitor pool. It has previously been speculated that the age-related enlargement of the HSC pool is related to impaired HSC differentiation and enhanced HSC self-renewal^39,40,44^. Considering NR did not significantly affect HSC cell-cycle dynamics, we hypothesise that the reduction in HSC numbers following NR is due to a more efficient exit from the stem cell pool via differentiation and lineage commitment. As the age-related enlarged stem and progenitor pool has been associated with an increased propensity for malignant transformation^66^, NR administration might also lower the risk for blood cancers.

In a transplant setting NR treatment significantly improved the ability of aged HSC to reconstitute the hematopoietic system of recipient mice, evidenced by increased donor contribution. However, the improved engraftment was only evident when both transplant donors and recipients were treated with NR. With these considerations, NR treatment might enable BM donations from individuals >45 years of age, the normal cut off age^6,7^, resulting in efficient engraftment in recipients in scenarios where younger donors are not available.

**Fig. 8:**
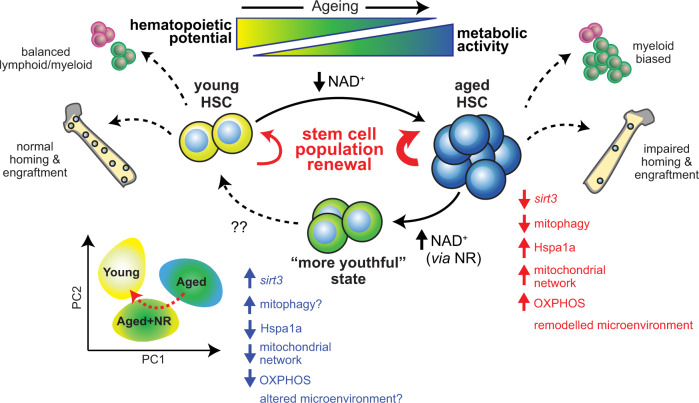
Proposed model of the effects of NR treatment on aged HSC. With age, HSC undergo several changes including expansion of the stem cell pool, reduction in homing and engraftment potential and myeloid biased differentiation. These functional changes are associated with reduced *Sirt3* expression and reduced mitophagy with a concomitant increase in the mitochondrial network and increased metabolism that is reliant on oxidative phosphorylation. These changes are underpinned by the altered transcriptional programme of aged HSC. Repletion of the age-associated decrease in NAD levels in HSC via nicotinamide riboside (NR) results in a reduction of the HSC pool as well as changes in the transcriptional programme of aged HSC towards a “more youthful” state and is associated with improved engraftment and partial reversion of the myeloid bias following transplantation of HSC isolated from NR-treated aged mice. These effects may be driven by increased *Sirt3* expression, reduced levels of Hspa1a and decreased metabolism through oxidative phosphorylation as well as increased mitophagy, which results in a reduction of mitochondrial network and output.

Our data points towards a model (Fig. 8) where NR treatment results in cell-autonomous, metabolic improvements which promote the functional benefits we observed. However, we cannot exclude that (1) metabolic improvements merely correlate with functional improvements rather than causing them and (2) the effects are potentially indirectly mediated by effects on a different cell type (e.g. bone marrow niche cells or cells responsible for the secretion of systemic factors).

Notwithstanding the restorative effects of NR treatment on metabolism, stem cell pool size, reconstitution potential and transcriptional signature, the myeloid differentiation bias was only partially corrected. While NR treatment resulted in a significant increase in the number of lymphoid progenitors and a concurrent significant decrease in myeloid progenitors following treatment of aged mice, this did not translate into a significantly altered number of lymphoid and myeloid cells under homeostatic conditions, or in a transplant setting. Differences in cell cycle and/or differentiation potential of HSC and committed progenitors from the NR-treated aged animals relative to young mice, together with an overall mild corrective effect of progenitor frequency, might be responsible for this discordance.

While NR treatment shifted aged HSC towards an intermediate state in-between young and old, it is not surprising that some functional aspects of HSC ageing, such as HSC homing, were not entirely correct. We propose that in order to achieve complete restoration of a youthful state in aged HSC, a multipronged approach will be required to complement NR-mediated metabolic restoration with additional therapeutic intervention to fully shift the molecular and functional signature of aged HSC to a young state. As such, a recent study has shown supplementation with mitoquinol (MitoQ), a mitochondrial-targeted coenzyme-Q10, can also improve the function and molecular signature of aged HSC, albeit via a different mechanism^67^. In contrast to NR treatment, which resulted in reduced mitochondrial mass in young^34^ and aged HSC (current study), MitoQ treatment did not affect mitochondrial content but instead modulated mitochondrial membrane potential. Although both treatment regimens significantly enhanced the engraftment potential of aged HSC and induced transcriptional signatures indicative of increased DNA repair potential, there were treatment-specific effects. At the transcriptional level, MitoQ treatment reduced the expression of genes associated with inflammation^67^, conversely, NR treatment reduced the expression of genes associated with mitochondrial stress (i.e. Sirt3 induction, Hspa1a downregulation). At the functional level, MitoQ treatment performed better at reducing the myeloid bias of aged HSC^67^, however, even after 5 months of treatment did not significantly shrink the age-enlarged HSC pool; which was achieved by NR treatment after a mere 2 months. Hence, in future studies, it will be interesting to explore whether NR and MitoQ treatment might have additive or even synergistic effects in improving aged HSC function.

In conclusion, we introduce NR treatment, a well-tolerated form of Vitamin B3, as a novel approach to completely revert a key hallmark of HSC ageing by re-establishing youthful metabolic potential. This restoration significantly improved many functional defects associated with HSC ageing. Accordingly, NAD repletion could form the basis of future clinical intervention strategies to ultimately fully correct aged HSC to levels not significantly different from young HSC. Further studies will be required to establish whether our findings can be extended to aged human HSC and whether different forms of Vitamin B3 have distinct effects on aged HSC as has been reported for young mouse HSC^34^. It will also be important to determine whether enhanced engraftment is stable if NR is eventually withdrawn after full hematopoietic reconstitution.

## Methods

### Mice

Young (6–12 weeks) and aged (20–24 months) C57BL/6, green fluorescent protein (GFP), red fluorescent protein (RFP), Vav1-iCre-blue fluorescent protein (BFP) and Vav1-iCre-Tomato mice of both genders were bred at Monash Animal Research Platform (MARP, Monash University, Clayton, Australia). The Vav-iCre (B6.Cg-Tg(Vav1-iCre)A2Kio/J) and tomato ((B6.Cg-Gt(ROSA)26Sortm 14 (CAGtdTomato) HzeJ)) mice were purchased from the Jackson Laboratory (Bar Harbor, Maine, USA) and crossed at MARP. For NR treatment experiments female mice were used. Wild type and the indicated ubiquitously fluorescently tagged mouse strains were used in transplant settings enabling the discrimination between donor and recipient derived cells. For transplant experiments, young mice of both genders were used as recipients. Mice received mouse chow and acidified water with or without NR (3 mg/ml) ad libidum as per a previous study^68^. Where mice received NR water, it was replaced every two days. All animal experiments were approved by the Monash Animal Services Ethics Committee and conducted in compliance with the specified ethics regulations.

### Fluorescence-activated cell sorting (FACS)-mediated purification of hematopoietic cell types

Bone marrow (BM) was isolated from young and aged mice as previously described^69^. Briefly, femurs, tibia and iliac crests were excised and the epiphyseal and metaphyseal regions removed. The bones were ground with a mortar and pestle in phosphate-buffered saline (PBS) 2% fetal calf serum (FCS) to liberate BM cells. Crushed bone fragments were digested with collagenase I (3 mg/ml; Worthington) and dispase II (4 mg/ml; Gibco) at 37 °C in an orbital shaker for 5 min, washed in PBS 2% FCS and filtered. The two cellular BM fractions were then combined, washed in PBS 2% FCS and filtered.

To isolate macrophages, whole-BM cells were stained with a cocktail of anti-mouse antibodies (Sca-1, cKit, B220, Ter119, CD3, Mac-1, Ly6C, Ly6G and F4/80) and Sca-1^−^cKit^−^Mac-1^+^B220^−^TER119^−^CD3^−^Ly6C^+^Ly6G^−^F480^+^ cells purified using flow cytometry. To isolate neutrophils, whole-BM cells were stained with a cocktail of anti-mouse antibodies (B220, Ter119, Mac-1, Ly6C, Ly6G) and B220-Ter119^−^Mac^+^Ly6C^+^Ly6G^+^ cells purified using flow cytometry. To isolate B cells whole-BM cells were stained with a cocktail of anti-mouse antibodies (B220, Ter119, Mac-1) and B220^+^Ter119^−^Mac-1^−^ cells purified using flow cytometry.

Mononuclear cells were obtained by density centrifugation of BM using Nycoprep (Axis-Shield) and lineage negative (Lin^−^) cells separated as previously described^70^. Briefly, low-density cells were stained with rat-anti-mouse B220 (CD45R), Mac-1 (CD11b), Gr-1 and Ter119 antibodies (BD Pharmingen, Franklin Lakes, NJ, US). Lin^−^ cells were obtained using anti-rat Dynal beads (Invitrogen) as previously described^71^, and stained with a cocktail of anti-mouse antibodies (Sca-1, cKit, CD48 and CD150) to isolate hematopoietic stem and progenitor cells. Cell sorting was performed on an Influx (BD) cell sorter using propidium iodide (PI, Sigma Aldrich) to exclude dead cells.

### Flow cytometry analysis and assays

Flow cytometric analysis was performed using an LSRII (BD Biosciences) as previously described^72^. Whole-BM cells were immunolabeled with a lineage cocktail (anti-B220, anti-CD3, anti-Gr1 and anti-Mac-1), anti-Sca-1, anti-cKit, anti-CD48, anti-CD150, anti-CD127, anti-Flt3, anti-CD34 and anti-CD16/32 antibodies. Secondary labelling was performed using streptavidin BUV805 where appropriate. Cells were analysed as HSPC (Lin^−^Sca-1^+^cKit^+^), HSC (Lin^−^Sca-1^+^cKit^+^CD150^+^CD48^−^), MMP (Lin^−^Sca-1^+^cKit^+^CD48^+^), granulocyte–macrophage progenitors (GMP; Lin^−^CD127^−^Sca-1^−^cKit^-^CD34^+^CD16/32^+^), lymphoid-primed multipotent progenitors (LMPP; Lin^−^Sca-1^+^cKit^+^CD34^+^Flt3^+^). Differentiated myeloid cells (Gr1/Mac-1^+^) and differentiated lymphoid cells (B220^+^, CD3^+^) on a LSRII (BD) as previously described^10,41,73^. To assess CD69 activation, whole-BM cells (200,000) were immunolabeled with anti-CD69, anti-B220, anti-Ly6G, anti-Ly6c, anti-Mac-1, anti-CD11c and F4/80. To assess cell cycle, whole-BM cells (2 × 10^6^) were immunolabeled with a lineage cocktail (anti-B220, anti-Gr1, anti-Mac-1), anti-Sca-1, anti-cKit, anti-CD48 and anti-CD150 antibodies, washed and then stained with anti-Ki67-BV786 (BD Biosciences) and Hoechst 33342 (20 μg/ml) using Fixation/Permeabilization Kit (ThermoFisher) according to the manufacturer’s instructions. A full list of antibodies and their working concentrations used in this work is detailed in Supplementary Table 1.

### Quantification of NAD levels

Quantification of relative NAD levels was performed on 1.5 × 10^5^ cells per sample (each coming from a pool of 3–5 mice) using the NAD^+^/NADH Quantification Colorimetric Kit by BioVision (Catalogue #K337) according to the manufacturer’s instructions.

### Transcriptional profiling of HSC

Ribonucleic acid (RNA) was extracted and purified from young and aged HSC (20,000–25,000 cells from individual mice) using the Direct-zol RNA micro kit (Zymo Research) according to the manufacturer’s instructions (RNA integrity number value >8).

For RNA sequencing a 3-prime biased approach was used to generate sequencing libraries. An 8 bp sample index and a 10 bp unique molecular identifier (UMI) were added during initial poly(A) priming and pooled samples were amplified using a template-switching oligonucleotide. The Illumina P5 (5′ AAT GAT ACG GCG ACC ACC GA 3′) and P7 (5′ CAA GCA GAA GAC GGC ATA CGA GAT 3′) sequences were added by PCR and Nextera transposase, respectively. The library was designed so that the forward read (R1) utilises a custom primer (5′ GCC TGT CCG CGG AAG CAG TGG TAT CAA CGC AGA GTA C 3′) to sequence directly into the index and then the 10 bp UMI. The reverse read (R2) uses the standard R2 primer to sequence the cDNA in the sense direction for transcript identification. Sequencing was performed on the NextSeq550 (Illumina), using the V2 High output kit (Illumina, #TG-160-2005) in accordance with the Illumina Protocol 15046563 v02, generating 2 reads per cluster composed of a 19 bp R1 and a 72 bp R2. Sample reads were aligned to the mouse genome (GENCODE GRCm38 primary assembly) using STAR version 2.4.2a^74^. Reads with duplicated UMIs were removed using the software Je option markdupes^75^. Transcript quantification was performed using feature Counts (exonic regions of GENCODE’s vM15 annotation version)^76^, and transcripts with more than 5 sequencing reads and 2 counts per million of mapped reads in at least two samples were used for further analysis. This resulted in a filtered count matrix of 16,154 genes. The resulting filtered count matrix was imported into edgeR and effective library sizes were calculated using the TMM method^77^.

To generate an overview between the similarities and differences between conditions and examine the consistency between replicate samples the count data were transformed into log(cpm) utilising the edgeR cpm function with a (pior.count = 10). Next, log(cpm) were used to generate a hierarchical clustering based on the sample-to-sample euclidean distances using the R *dist* and hclust (method = “ward.D2”) functions, and a PCA plot to display the samples based on their two first principal components, using the DESeq2 package plotPCA function^78^.

Differentially expressed genes were identified using the edgeR pipeline^79,80^. In brief, library size normalisation was performed using the TMM method^81^, estimates of a common negative binomial dispersion, the abundance-dispersion trend by Cox–Reid profiled likelihood and empirical Bayes estimate of the negative dispersion parameter per gene were calculated followed by the fitting of the negative binomial generalised log-linear model to the read counts^80^. Differential expression was tested using likelihood ratio tests. The resulting *P*-values were corrected for multiple testing with Benjamini–Hochberg to control for the false discovery rate (FDR). Only genes with FDR ≤ 0.05 were considered as differentially expressed.

Differentially expressed genes were imported into DAVID^82^ to identify general gene ontology categories. Expression heatmaps were generated using Multiple Experiment Viewer (MeV)^83^ using the log(cpm) values and centred by their gene median expression across samples.

Differentially expressed genes were imported into STRING-DB^48^ to determine gene interactions and metabolic gene ontologies. The STRING gene network was imported and visualised into Cytoscape 3.7.1 using the stringApp 1.5.1^84^. This list of gene interactions was exported and reformatted into a Simple Interaction File and its gene network determined using CYTOSCAPE^85^ as per a previous publication^86^. The subsets of genes involved in the metabolic biological processes of interest were also exported from STRING-DB and overlaid onto the Cytoscape gene network using CYTOSCAPE. The option “Pie” in “Image/Chart” was used.

The 2635 differentially expressed genes from the previously published dataset^45^ were passed from mm9 coordinates to m10 coordinates using the liftOver tool^87^ resulting in 2632 mm10 regions. Next, we overlapped the mm10 coordinates to (GENCODE’s vM15 annotation version) using bedtools intersect^88^ resulting in the overlap of 2614 genes of which 2464 were found in our filtered count matrix (16,154 genes). The resulting overlap between 2464 differentially expressed genes from Sun et al.^45^ and the 3338 differentially expressed genes in this study resulted in an overlap of 1133 genes between both datasets. The overlap between gene lists was statistically evaluated by calculating the hypergeometric test using the R *phypher* function. The number of genes passing the initial filtering cutoff, 16,154, was considered as the total number of genes.

Gene set activity analysis for positive regulators of mitophagy (via GO:0000423 from The Jackson Laboratory’s Mouse Genome Informatics database: *Ambra1*, *Atg13*, Cdc37, *Clec16a*, *Hdac6*, *Huwe1*, *Optn*, *Pink1*, *Smurf1*, *Sqstm1*, *Trp53*, *Vps13c*, *Vps13d*) was performed as follows: For each sample, the activity of a gene set (gene signature) was approximated by the area under the recovery curve, which was constructed based on the cumulative number of genes in the gene set that was present in the top 15% most highly expressed genes^89^. A higher score suggests the sample contains not only more genes in the top 15% gene expression, but more genes at the higher expression end of these 15% genes, and thus indicates a higher activity of the gene signature.

### Analysis of mitochondrial respiration

Mitochondrial activity of freshly isolated progenitor cells, stem cells and macrophages, neutrophils and B cells was measured using a seahorse metabolic influx assay according to the manufacturer’s instructions (Agilent Technologies). Optimisation runs identified the ideal number of cells per well for the respective cell types using the 96-well plate assay format (10,000–25,000 HSPC and HSC/well, 25,000 macrophages/well, 50,000 neutrophils/well, 100,000 B cells/well). Cells were re-suspended in serum-free seahorse XF DMEM medium (pH 7.4 supplemented with 25 mM glucose and 1 mM pyruvate) and plated onto a 96-well plate pre-coated for 1 h with Cell-Tak (22.4 μg/ml, Corning). Plates were immediately analysed using a seahorse Bioscience XFe96 extracellular flux analyser (Agilent Technologies). The oxygen consumption rate (OCR) was measured at 37 °C under basal conditions and in response to 1 μM oligomycin, followed by 2 μM fluoro-carbonyl cyanide phenylhydrazone (FCCP) and 1 μM retone/antimycin A (all from Agilent Technologies). The OCR from cells was normalised against the total protein concentration of cells measured by a DC protein assay (Bio-Rad) according to the manufacturer’s instructions. Every seahorse assay was run with the following instrument settings: 7 min mix time and 4 min read time.

### Analysis of mitochondrial network size

Freshly isolated HSC were seeded on µ-Slide 8-wells (Ibidi, 80826) pre-coated for 30 min with Cell-Tak (22.4 μg/ml, Corning) before being fixed in 4% paraformaldehyde /Sorensen’s buffer for 10 min and washed with water. After permeabilization with 0.5% (w/v) Triton X-100 in PBS, cells were washed twice using PBS with 0.2 % (w/v) Tween-20 and incubated with anti-Tom20 (1:500; Santa Cruz, SC-11415) for 90 min at room temperature. After two wash steps, the cells were labelled with Alexa-Fluor-564 conjugated anti-rabbit-IgG (Molecular Probes, A-11011) followed by Hoechst 33342 (1 µg/ml). For imaging, confocal microscopy was performed on a Leica TCS SP8 inverted confocal microscope equipped with HyD detectors using a ×63/1.40 NA oil immersion objective (HC PLAPO, CS2, Leica Microsystems). 3× line accumulation, 600 Hz bi-directional scanning speed and 25% Smart Gain were used as a default setting for the image acquisition with HyD detectors. Microscopy data were recorded using the Leica LAS X Life software (v3.5.5.19976). Images in all experimental groups were obtained using the same settings. *Z*-sectioning was performed using 300-nm slices. Leica.lif files were converted to multi-colour.tiff composite stacks using custom-written Fiji/ImageJ macros (v1.52n)^90^. For 2D image analysis, the mitochondrial image stack was filtered with a Gaussian Blur (2 pixel) and converted to binary image files using the ‘Moments’ Thresholding method. Those segmented image objects were analysed using the “Analyse Particles” function in Fiji/ImageJ, and the fluorescently stained area per cell was quantified. For volumetric analysis, confocal *z*-stacks of Tom20-labelled mitochondria were mean filtered (1px) and then segmented in 3D using the “3D Watershed method” (Seed Threshold: 40; Image Threshold: 30; Image&Seed data: *Image input file to be segmented*; radius:1) of the 3D ImageJ Suite (https://imagejdocu.tudor.lu/plugin/stacks/3d_ij_suite/start)^91^. Segmented 3D objects were loaded into 3D ROI manager and quantified using “Measure 3D”. The resulting volume measurements were exported and saved as.xlsx files for further downstream processing. If necessary, size-filtering was performed to exclude debris or non-specific staining. Average volumes of all mitochondrial objects per cell (unit: pixels^3^) plotted in GraphPad Prism. Surface volume renderings of mitochondrial objects were created using Fiji/ImageJ (v1.52n). Confocal *z*-stacks of mitochondrial staining were loaded into Fiji/ImageJ and visualised using the 3D viewer plugin (Threshold: 50; Resampling Factor: 2). The background was set to white and views exported as.tiff files.

### Electron microscopy

Cell suspensions were incubated on Poly-l-lysine-coated dishes for 15 min then fixed with 2.5% glutaraldehyde (Electron Microscopy Services) in PBS for 30 min at room temperature.

Samples were then further fixed and contrasted with 1% osmium tetroxide (ProSciTech) then 2% uranyl acetate (ProSciTech). Cells were serially dehydrated in increasing concentrations of ethanol, then embedded in LX-112 resin (Lad Research) using a Pelco Biowave. Samples were polymerised in a 60 °C oven for 24 h, then ultrathin sections were obtained using a Leica Ultracut UC6 Ultramicrotome. Sections were viewed and micrographs acquired using a JEOL JEM-1011 transmission electron microscope equipped with a Morada CCD camera (Olympus) under the control of iTEM software.

### Mitotracker green assay

Whole-BM cells were immunolabeled with a lineage cocktail (anti-B220, anti-CD3, anti-Gr1 and anti-Mac-1), anti-Sca-1, anti-cKit, anti-CD48, anti-CD150 for 20 min at 4 °C, washed and dry pelleted. The cells were re-suspended in 100 μl of PBS 2% FCS containing verapamil (50 μM), incubated for 30 min at 37 °C and without washing was then treated with 100 nM (final) of mitotracker green for a further 30 min at 37 °C. Propidium iodide was spiked directly into the cell suspensions and samples were analysed on a LSRII as described above.

### Western blot

Bone marrow Lin^−^Sca-1^+^cKit^+^ (LSK) cells were sort purified by FACS as described above and lysed for 20 min on ice using RIPA buffer (Pierce RIPA buffer containing 1× Halt Protease and Phosphatase Inhibitor cocktail, Thermo Scientific #89900 and #1861281). The samples were centrifuged at 18,000×*g* for 10 min and the supernatants were collected and frozen at −80 °C. Each sorted young sample is comprised of a pool of 5 young mice, while aged and NR-treated aged samples are obtained from individual mice. The lysates were thawed, prepared and loaded onto the JESS automated Western Blot system (ProteinSimple) according to the manufacture and analysed for Hspa1a/Hsp70 (Cell Signalling; rabbit polyclonal #4872) on the chemiluminescence channel and vinculin (Sigma; mouse monoclonal #V9264) as loading control on the near-IR channel. The data was analysed using Compass software (https://www.proteinsimple.com/compass/downloads) and the area under the curve (AUC) was calculated from the electropherograms. The Hspa1a signal was then quantified relative to the vinculin loading control for each sample.

### Homing assay

Sorted HSC from C57BL/6 mice were labelled with the fluorescent tracking dye carboxy fluorescein succinimidyl ester (CFDA-SE, CFSE when incorporated, Molecular Probes, Eugene OR, US) or seminaphtorhodafluor-1-acetoxymethylester (SNARF-1; Invitrogen) to use as donor cells as previously described^71,72^. In other experiments, sorted HSC from GFP or RFP mice were used as donor cells. A total of 7000–50,000 donor cells were transplanted into each non-irradiated C57BL/6 recipient via lateral tail vein injection with an additional non ablated 200,000 whole-BM carrier cells. The homing efficiency of donor HSC was assessed after 15 h as previously described^72^. In brief, femurs, tibia and iliac crests were removed, BM isolated and the content of CFSE^+^, SNARF^+^, GFP^+^ or RFP^+^ cells analysed by flow cytometry.

### Engraftment assay

#### In vivo NR-treated aged HSC into NR-treated recipients

To assess BM reconstitution potential, HSC from aged (100 cells) or NR-treated aged (100 cells) C57BL/6 wild-type mice were co-transplanted with 30 HSC from young RFP mice together with 200,000 irradiated (15 Gy) whole-BM carrier cells into irradiated (9.5 Gy) young GFP or BFP recipients. The recipients transplanted with NR-treated aged HSC were supplemented with acidified water with NR (3 mg/ml), and the recipients transplanted with aged HSC were provided with acidified water without NR, ad libidum.

#### In vitro NR-treated aged HSC into NR-treated recipients

Tomato^+^ HSC were sorted from aged Vav1-iCre-Tomato donor mice (*n* = 3) into a 96-well plate at 450 HSC/well in 50 μl of StemSpan SFEM II (10 ng/ml rmSCF and 10 ng/ml rhFLT3L) with and without 1 mM NR. In addition, GFP^+^ HSC were sorted from young GFP mice (*n* = 6 pooled) at 150 HSC/well into SFEM II (10 ng/ml rmSCF and 10 ng/ml rmFLT3L). The plates were incubated in a 5% O_2_ triple-mix incubator at 37 °C for 2 days. The wells were pooled to allow transplant of 450 tomato^+^ aged HSC and 150 young GFP^+^ HSC plus 200,000 non-irradiated whole-BM carrier cells into each young irradiated C57Bl/6 recipient. Five recipients were transplanted for each untreated aged (*n* = 3; 15 recipients total) and aged NR-treated donor group (*n* = 3; 15 recipients total). The recipients transplanted with NR-treated aged HSC were supplemented with acidified water with NR (3 mg/ml), and the recipients transplanted with untreated aged HSC were provided with acidified water without NR, ad libidum.

#### In vivo NR-treated aged HSC into untreated recipients

To assess engraftment into recipients that were not treated with NR, sorted GFP^+^ HSC (300 cells) from aged or NR-treated aged GFP mice were transplanted with 200,000 irradiated (15 Gy) whole-BM carrier cells into irradiated (9.5 Gy) young C57BL/6 wild-type recipients. The recipients were provided with acidified water, ad libidum.

#### Untreated aged HSC into NR-treated recipients

To assess the engraftment of aged HSC into treated NR, sorted GFP^+^ HSC (300 cells) from aged GFP mice (*n* = 2 pooled) and RFP^+^ HSC (100 cells) from young RFP mice (*n* = 8 pooled) were transplanted with 200,000 irradiated (15 Gy) whole-BM carrier cells into irradiated (9.5 Gy) young C57BL/6 wild-type recipients. The recipients were provided with either acidified water supplemented with NR (3 mg/ml) (*n* = 9) or water without NR (*n* = 8), ad libidum.

Engraftment levels were assessed at 6, 12, and 20 weeks post-transplant for PB and 20 weeks post-transplant for BM. Multi-lineage reconstitution and the frequency of different donor-derived populations were examined by staining with a lineage cocktail (anti-B220, anti-CD3, anti-Gr1 and anti-Mac-1), anti-Sca-1, anti-cKit, anti-CD48, anti-CD150, anti-CD127, anti-Flt3, anti-CD34 and anti-CD16/32 antibodies. Secondary labelling was performed using streptavidin BUV805 where appropriate. Cells were analysed as HSPC, HSC, GMP, LMPP, myeloid cells (Gr1/Mac-1^+^) and lymphoid cells (B220^+^, CD3^+^) on a LSRII (BD) as previously described^10,41,73^.

### Statistical analysis

Data were assessed for normal distribution with a Shapiro–Wilk normality test using GraphPad Prism 8 (GraphPad Software Inc, San Diego, USA) and presented as mean ± standard error of the mean (s.e.m) or as box-and-whisker plots, Box indicates 25th to 75th percentile, the line indicates the median and whiskers indicate min to max values. Differences between means were evaluated by an unpaired two-sided Student’s *t-*test or one-way ANOVA with post-hoc multiple comparisons test (Tukey’s multiple comparison test) if data were normally distributed and the variance was equal. Non-parametric Mann–Whitney or Kruskal–Wallis test (with Dunn’s multiple comparison’s analysis) were used if data were not normally distributed or if the unequal variance between groups were observed. Two-way ANOVA with Geisser-Greenhouse correction and post-hoc multiple comparisons test (Holm–Sidak multiple comparisons test) was also used where appropriate. The difference between groups is considered statistically significant if *P* < 0.05.

### Reporting summary

Further information on research design is available in the Nature Research Reporting Summary linked to this article.

## Supplementary information

Supplementary Information

Description of Additional Supplementary Files

Supplementary Data 1

Supplementary Data 2

Supplementary Data 3

Reporting Summary

## Data Availability

Custom computer code used to generate results reported in the paper are available from the corresponding authors upon reasonable request.

## Source data

Source Data

## Data availability

The RNA Seq data have been placed in a public repository with the GEO accession number GSE165982. Additional data that support the findings of this study are available from the corresponding authors upon reasonable request. Source data are provided with this paper.
