# Supplementary material for: Nicotinamide riboside attenuates age-associated metabolic and functional changes in hematopoietic stem cells
Source: Nat Commun. 2021 May 11;12:2665. doi: 10.1038/s41467-021-22863-0 (PMC8113506; doi:10.1038/s41467-021-22863-0)
Supplement: Supplementary file 1 — Supplementary Information [file 41467_2021_22863_MOESM1_ESM.pdf]

# SUPPLEMENTARY INFORMATION

**Supplementary Fig. 1: Representitive flow cytometry plots for lymphoid and myeloid lineage cells.**

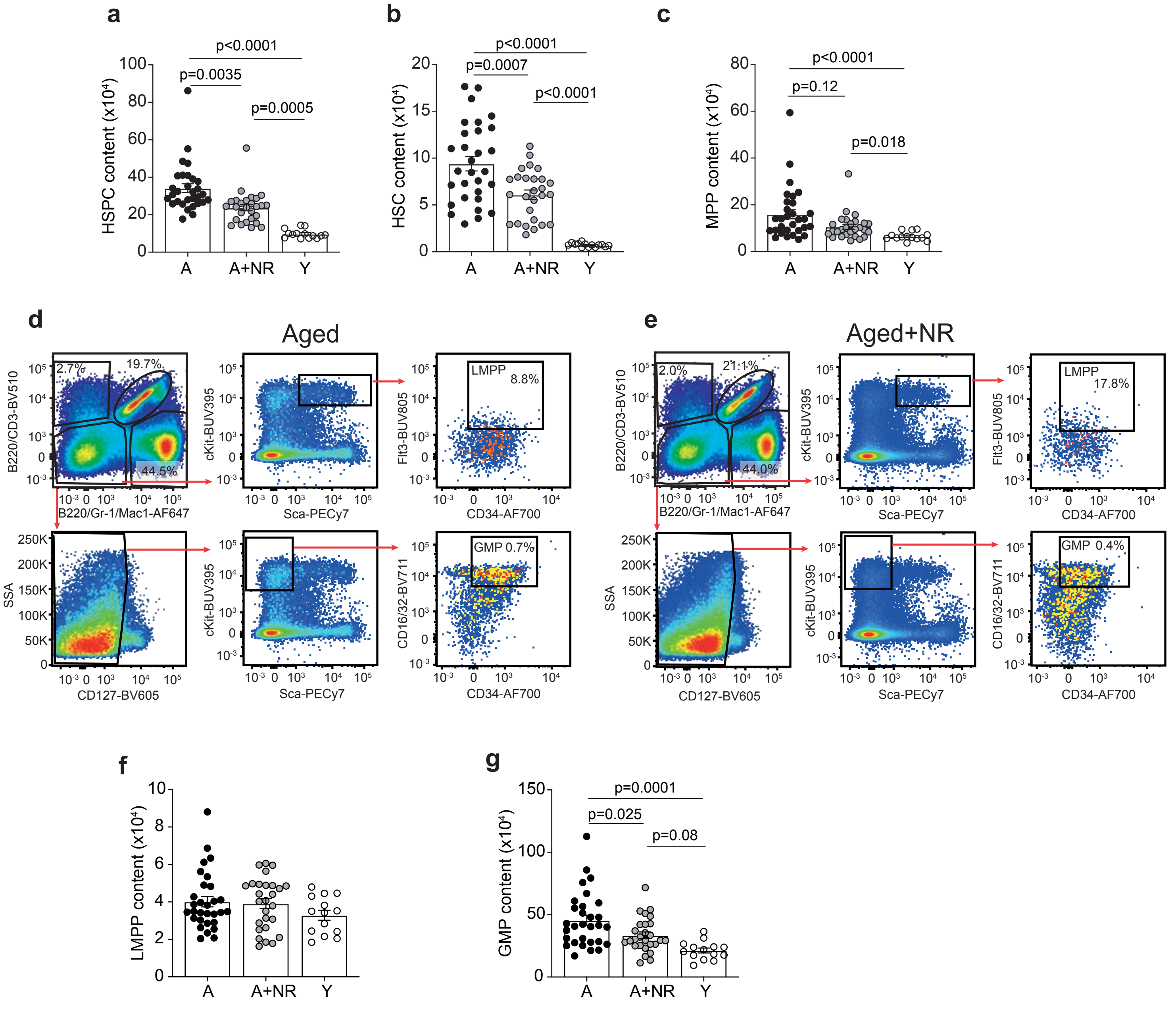

**Supplementary Fig. 1: Representative flow cytometry plots for lymphoid and myeloid lineage cells.** Number of **(a)** HSPC (Lin$^-$Sca-1$^+$cKit$^+$), **(b)** HSC (Lin$^-$Sca-1$^+$cKit$^+$CD150$^+$CD48$^-$) and **(c)** MPP (Lin$^-$Sca-1$^+$cKit$^+$CD48$^+$) per hindlimb in aged (n=30, biological replicates), NR treated aged (n=27, biological replicates) and young (n=14, biological replicates) mice. **(d)** Representative flow cytometry plot for LMPP (Lin-Sca-1$^+$cKit$^+$CD34$^+$Flt3$^+$) and GMP (Lin$^-$CD127$^-$Sca-1$^-$cKit$^+$CD34$^+$CD16/32$^+$) in aged **(e)** and **(f)** NR treated aged BM. **(f, g).** Number of **(f)** LMPP (Lin$^-$Sca-1$^+$cKit$^+$CD34$^+$Flt3$^+$) and **(g)** GMP (Lin$^-$CD127$^-$Sca-1$^+$cKit$^+$CD34$^+$CD16/32$^+$) in aged (n=30, biological replicates), NR treated aged (n=27, biological replicates) and young (n=14, biological replicates) BM. Data are mean ± s.e.m and is a pool from 8 separate collection days. Statistical analyses were performed using Kruskal-Wallis test, $p<0.0001$ (overall for **a**), $p<0.0001$ (overall for **c**) with individual groups compared using Dunn's multiple comparisons test, p-values indicated for **(a, c)** and Ordinary One-way ANOVA, $p<0.0001$ (overall for **b**), $p=0.25$ (overall for **f**), $p=0.0001$ (overall for **g**) with individual groups compared using Tukey's multiple comparisons test, p-values indicated for **(b, f, g).** Abbreviations: NR=nicotinamide riboside, BM=bone marrow, WBM=total bone marrow, Y=young, A=aged, A+NR = NR treated aged animal, Y+NR = NR treated young animal, HSPC=hematopoietic stem and progenitor cells, HSC=hematopoietic stem cells, MMP= multipotent progenitors, GMP=granulocyte–macrophage progenitors, LMPP= Lymphoid-primed multipotent progenitors. Source data are provided as a Source Data File.

**Supplementary Fig. 2: NR administration does not affect BM cellular composition in young mice.**

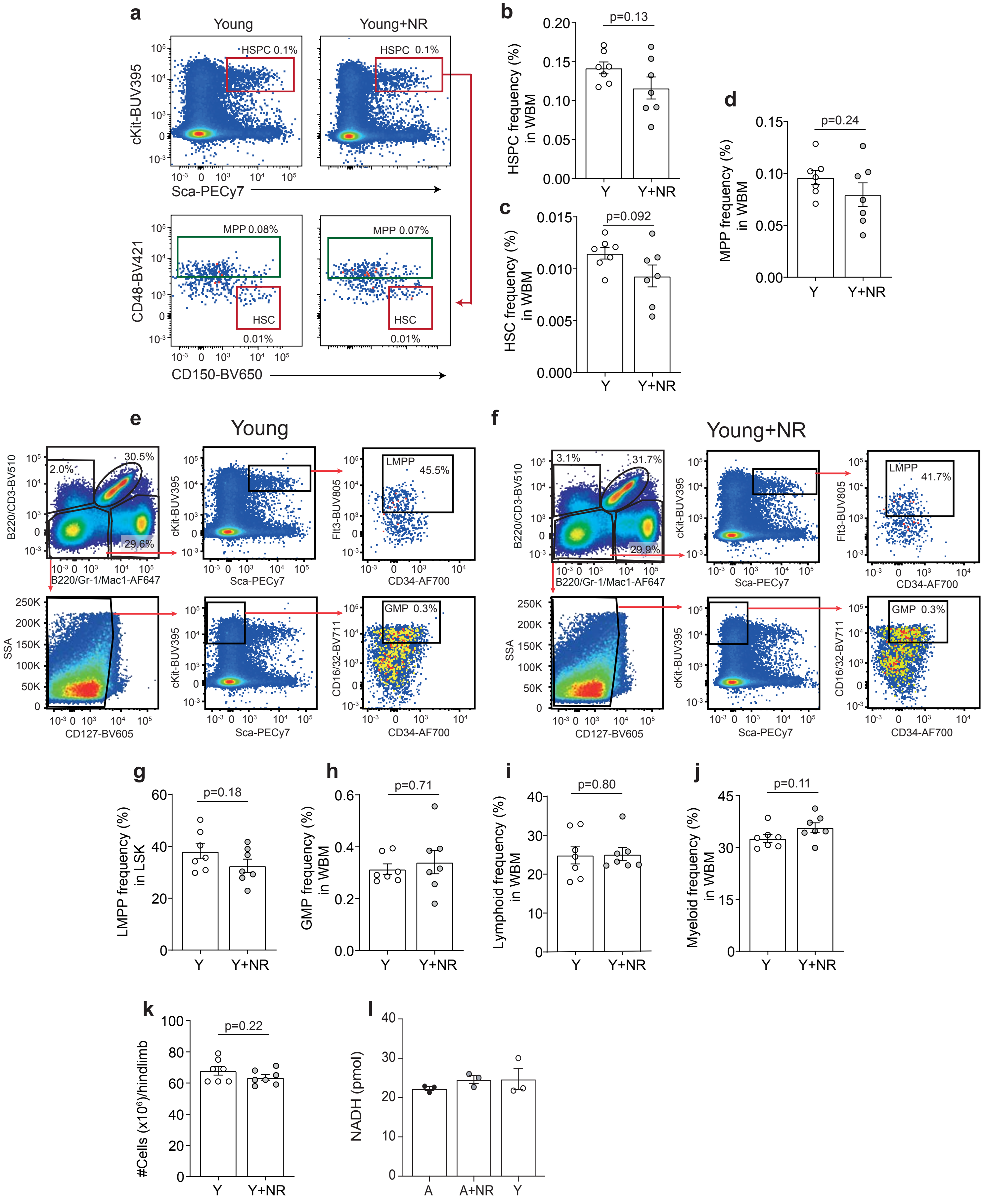

**Supplementary Fig. 2: NR administration does not affect BM cellular composition in young mice. (a)** Representative flow cytometry plots for HSPC, HSC and MPP in the BM of young controls and NR treated young mice (NR was administered for 8 weeks). **(b-d)** Incidence of **(b)** HSPC, **(c)** HSC, and **(d)** MPP in the BM of young (n=7, biological replicates) and NR treated young (n=7, biological replicates) mice. **(e, f)** Representative flow cytometry plots for LMPP and GMP in the BM of **(e)** young controls and **(f)** NR treated young mice (NR was administered for 8 weeks). **(g)** LMPP, **(h)** GMP, **(i)** lymphoid cells, and **(j)** myeloid cells. **(k)** Hindlimb BM cellularity in young and NR treated young mice. Data is mean ± s.e.m and statistical analyses were performed using an unpaired two-sided Student's t-test (**b-d, g, j, k**) or two-sided Mann-Whitney U-test (**h, k**), p-values indicated. **(l)** NADH levels are not significantly different in aged (n=3, biological replicates), NR treated aged (n=3, biological replicates) and young (n=3, biological replicates) mice. Data is mean ± s.e.m and statistical analysis was performed using Ordinary One-Way ANOVA, p=0.54 (overall for **l**). Abbreviations: HSPC=hematopoietic stem and progenitor cells, HSC=hematopoietic stem cells, BM=bone marrow, WBM=total bone marrow, NR=nicotinamide riboside, MMP= multipotent progenitors, GMP=granulocyte macrophage progenitors, LMPP=lymphoid-primed multi-potent progenitors, Y=young, Y+NR=NR treated young animal. Source data are provided as a Source Data File.

## Supplementary Fig. 3: Age and NR treatment related transcriptional changes in HSC.

**a**

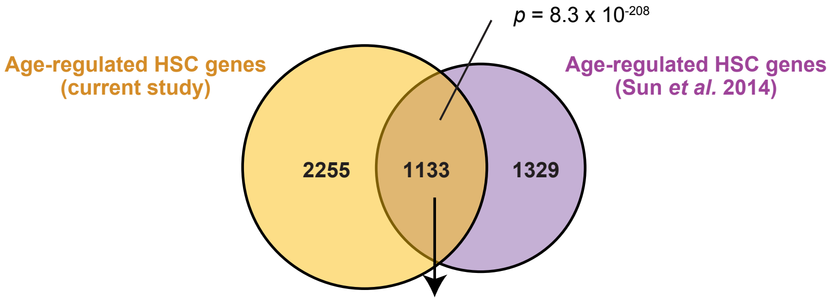


**Top GO categories**
Transcription ($p$ = 1.8 x $10^{-8}$)
DNA replication ($p$ = 2.7 x $10^{-8}$)
Response to Interferon-gamma ($p$ = 4.5 x $10^{-6}$)
Cell Proliferation ($p$ = 7.8 x $10^{-6}$)
Response to drug ($p$ = 1.2 x $10^{-5}$)

**b**

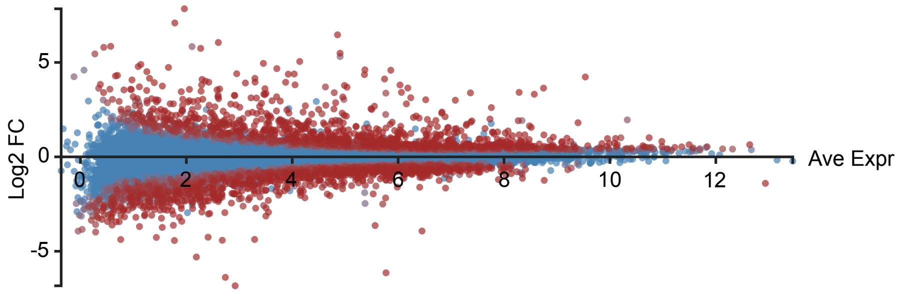


Differentially regulated **Young vs Aged HSC** (3388 genes)

**c**

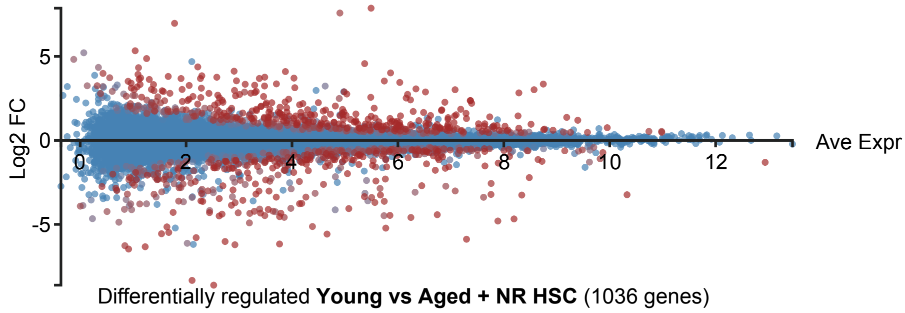


Differentially regulated **Young vs Aged + NR HSC** (1036 genes)

**Top GO categories**

Immune System process ($p$ = 7.4 x $10^{-17}$)
Cellular Response to Interferon-beta ($p$ = 9.3 x $10^{-12}$)
Innate immune response ($p$ = 1.7 x $10^{-8}$)
Response to drug ($p$ = 2.9 x $10^{-8}$)
Cell Proliferation ($p$ = 2.6 x $10^{-7}$)

**d**

FDRs associated with Figure 3b-e

| | Y vs A | A vs A+NR | Y vs A+NR |
|---|---|---|---|
| **HK2** | 9.05785e-06 | 0.039721882 | 0.170664841 |
| **FoxK1** | 0.005906023 | 0.021241006 | 0.932125149 |
| **Acox1** | 0.003769787 | 0.001399394 | 0.8329287 |
| **Idh1** | 0.298535234 | 0.002688513 | 0.127349496 |
| **Cox7a2l** | 0.0035829 | 0.005422534 | 0.975072219 |
| **Ndufc2** | 0.023085378 | 0.011155765 | 0.843931029 |
| **CD38** | 1.16e-07 | 0.00473744 | 0.150528706 |
| **Sirt3** | 0.241557211 | 0.021811671 | 0.467239704 |

**Supplementary Fig. 3: Age and NR treatment related transcriptional changes in HSC.** **(a)** Visualisation of the overlap of differentially expressed genes in this study between young and aged HSC (false discovery rate <0.05) was compared with the differentially genes from a published study[45]. A hyper geometric test was used to calculate significance of the overlap. DAVID was used to determine the top gene ontology categories associated with the 1133 differentially expressed genes shared by both data sets. **(b, c)** MA-plots visualising the differentially expressed genes **(b)** between young and aged HSC (false discovery rate <0.05) and **(c)** young and aged HSC from NR treated mice (false discovery rate <0.05). For panel **c** top gene ontology categories (DAVID) for differentially regulated genes are indicated. **(d)** Exact FDR values comparing aged (n=5, biological replicates), NR treated aged (n=5, biological replicates) and young HSC (n=5, biological replicates) for metabolism genes associated with Figure 3b-e. Statistical analyses were performed using R *phypher* **(a)**, DAVID **(a, c)** and EdgeR (false discovery rates between 2 groups were determined considering the third group as background, **a-d**). All data are mean ± s.e.m. Abbreviations: HSC=hematopoietic stem cells, NR=nicotinamide riboside, p=p-value.

## Supplementary Fig. 4: NR treatment impacts cell cycle.

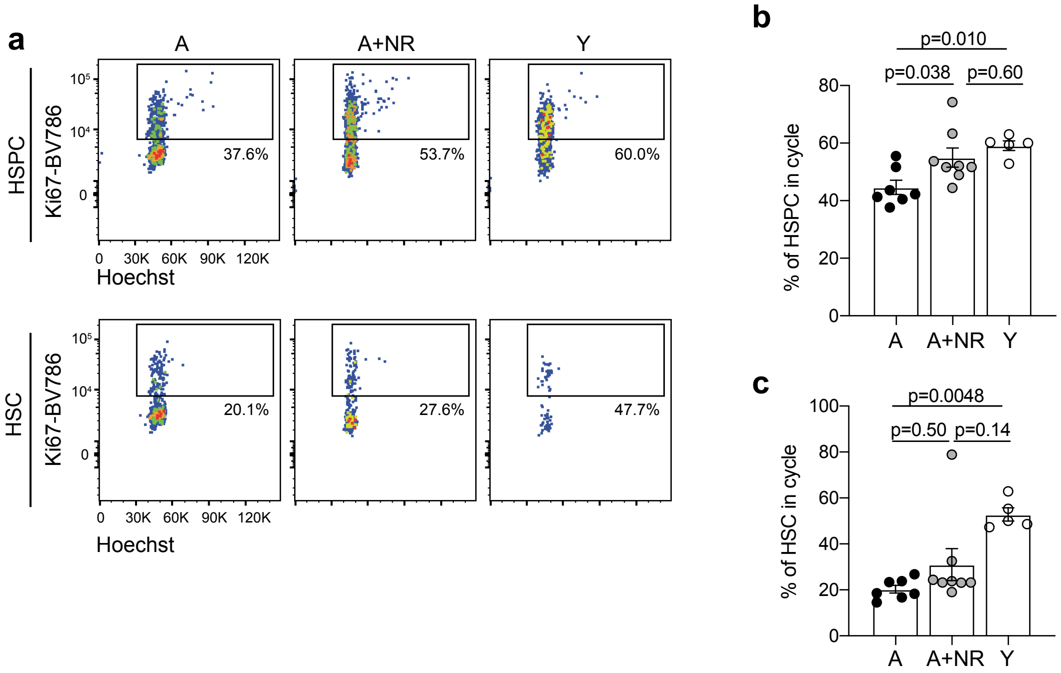

**Supplementary Fig. 4: NR treatment impacts cell cycle. (a)** Representative flow cytometry plots visualising percentage of HSPC and HSC that are in cell cycle ($Ki67^+$) in young (n=5, biological replicates), aged (n=7, biological replicates) and NR treated aged (n=8, biological replicates). **(b, c)** Quantification of percentage of HSPC and HSC in cycle. Data is mean ± s.e.m and statistical analyses were performed using Ordinary One-Way ANOVA, p=0.0084 (overall for **b)** with individual groups compared using Tukey's multiple comparisons test and Kruskal-Wallis, p=0.0026 (overall for **c**) with individual groups compared using Dunn's multiple comparisons test, p-values indicated. Abbreviations: NR=nicotinamide riboside, HSPC = hematopoietic stem and progenitor cells, HSC=hematopoietic stem cells, Y=young, A=aged, A+NR=NR treated aged animal. Source data are provided as a Source Data File.

**Supplementary Fig. 5: NR administration influences mitochondrial metabolism in young HSPC.**

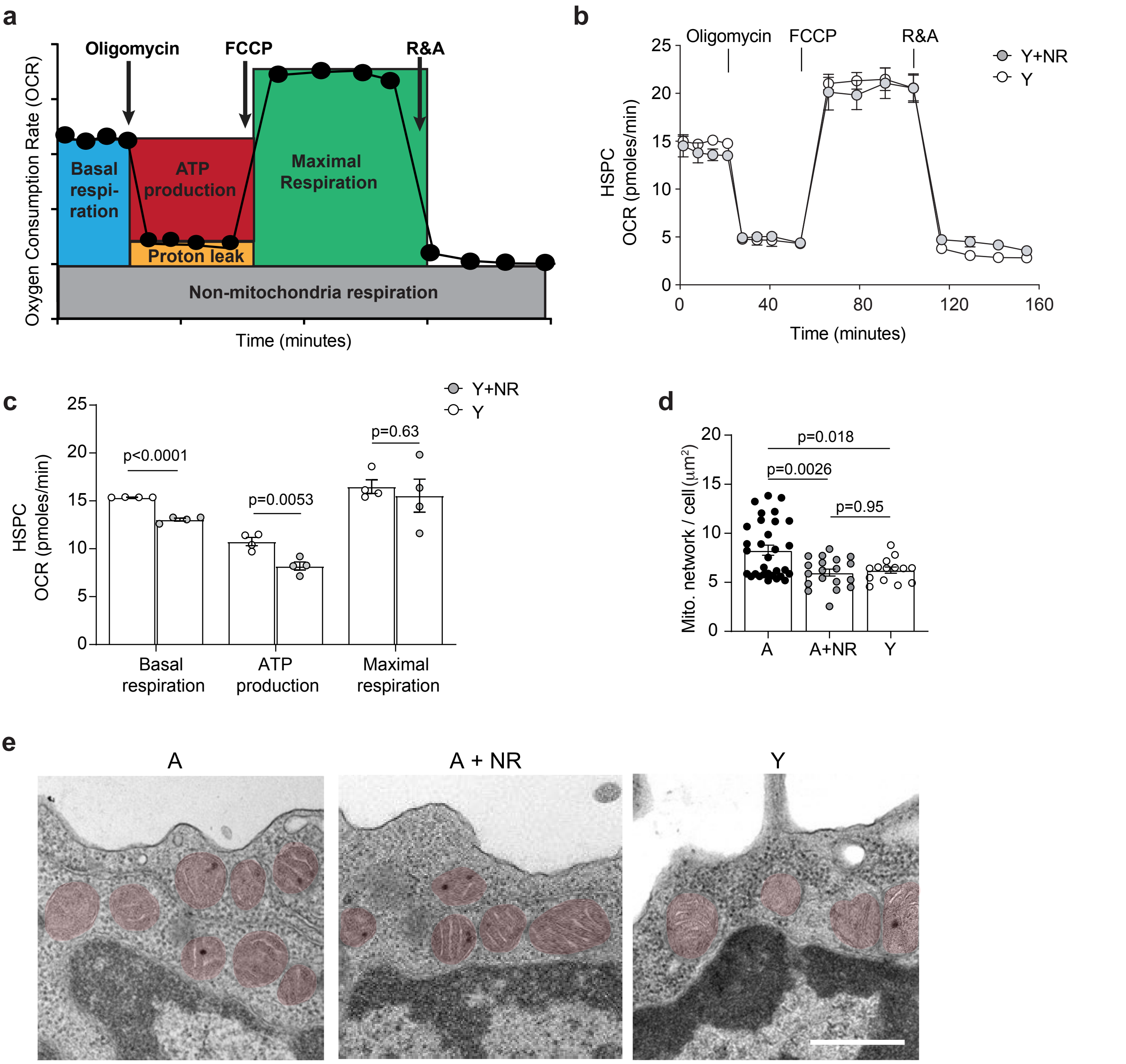

**Supplementary Fig. 5: NR administration influences mitochondrial metabolism in young HSPC. (a)** Schematic representation of the seahorse metabolic flux assay in live cells. At specific time points the ATPase inhibitor oligomycin, the uncoupling reagent FCCP and the inhibitors of the electron transport chain R&A are added, as indicated by arrows, to determine effects on oxygen consumption rate (OCR). Basal respiration is determined as average OCR before addition of oligomycin; ATP-linked respiration is calculated as the average difference in OCR before and after the addition of oligomycin relative to basal respiration; maximal respiratory rate is determined by subtracting the average OCR after addition of R&A from the average OCR after the addition of FCCP/before the addition of R&A. **(b)** Quantification of OCR in freshly isolated HSPC from young (n=4, biological replicates) and NR treated young (n=4, biological replicates) mice. **(c)** Basal respiration, ATP production and maximal respiration levels in young (n=4, biological replicates) and NR treated young (n=4, biological replicates) HSPC. Data are mean ± s.e.m and statistical analyses were performed using two-sided Student's t-test, p-values indicated for **(c)**. **(d)** 2D quantification of total cell mitochondrial network size ($\mu m^2$) / HSC isolated from aged (n=7, biological replicates), NR treated aged (n=5, biological replicates) and young (n=4, biological replicates) mice, two experiments with ~15–20 HSC quantified from each biological sample in each experimental repetition. Data is mean ± s.e.m. and is a pool across 2 collection days. Statistical analysis was performed using Ordinary One-way ANOVA, p=0.0012 (overall) with individual groups compared using Tukey's multiple comparisons test, p-values indicated for **(d)**. **(e)** Representative electron microscopy images of HSC isolated from aged (n=3, biological replicates), NR treated aged (n=3, biological replicates) and young (n=3, biological replicates) mice. Data was obtained from samples isolated across 3 collection days. The micrograph is representative of 10 images/cell per biological replicate for each experiment group; mitochondria are highlighted in red. Scale bar represents 500 nm

Abbreviations: NR=nicotinamide riboside, HSPC=hematopoietic stem and progenitor cells, HSC=hematopoietic stem cells, Y=young, A=aged, Y+NR= NR treated young animal, A+NR=NR treated aged animal. Source data are provided as a Source Data File.

**Supplementary Fig. 6: NR administration does not impact metabolism of committed cells.**

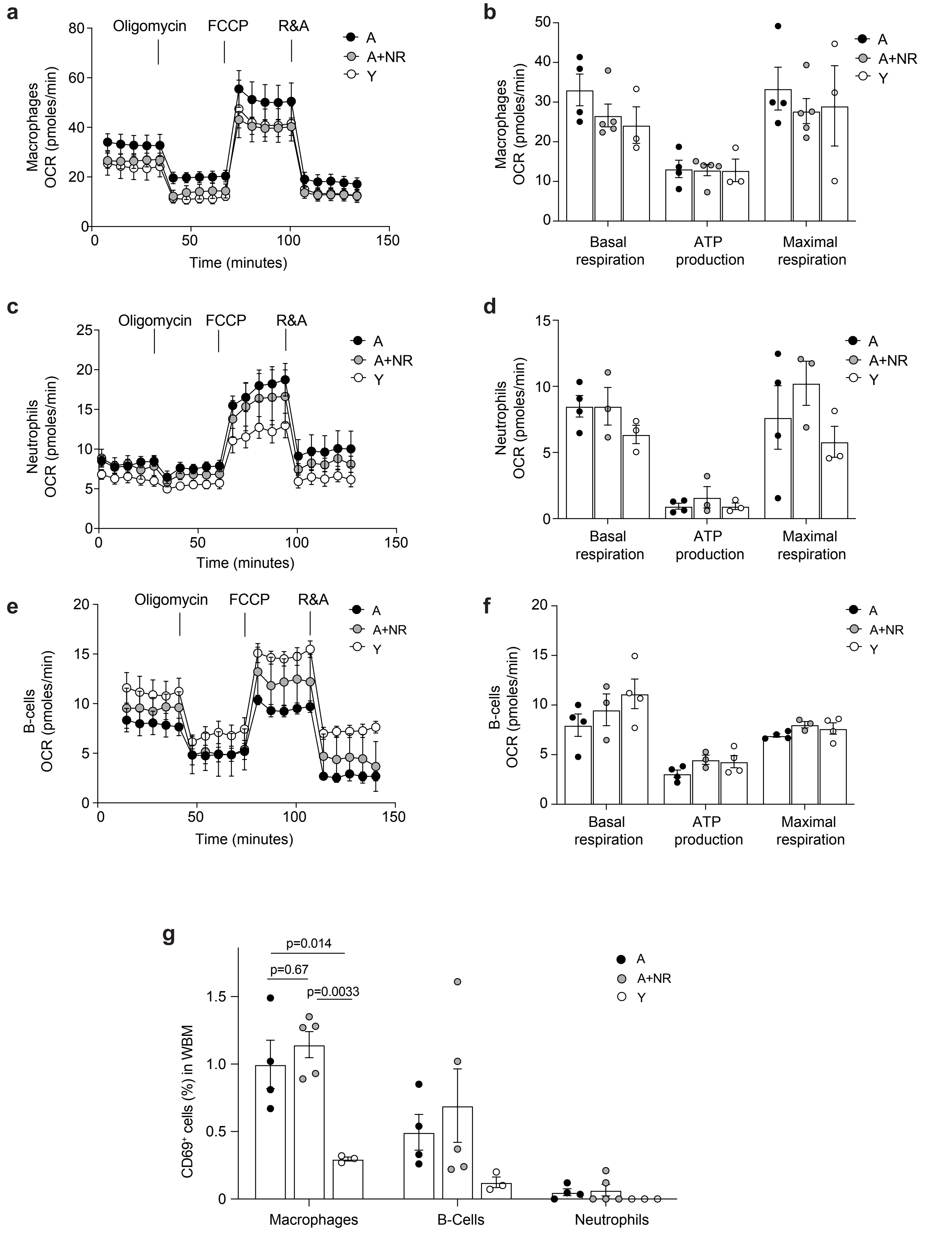

**Supplementary Fig. 6: NR administration does not impact metabolism of committed cells. (a)** Quantification of OCR in freshly isolated macrophages from aged (n=4, biological replicates), NR treated aged (n=5, biological replicates) and young (n=3, biological replicates) mice. **(b)** Basal respiration, ATP production and maximal respiration levels in aged, NR treated aged and young macrophages. Data is mean ± s.e.m and statistical analyses was performed using Kruskal-Wallis, p=0.14 (overall for basal respiration), p=0.93 (overall for ATP production), p=0.58 (overall for maximal respiration) **(c)** Quantification of OCR in freshly isolated neutrophils from aged (n=4, biological replicates), NR treated aged (n=4, biological replicates) and young (n=3, biological replicates) mice. **(d)** Basal respiration, ATP production and maximal respiration levels in aged, NR treated aged and young neutrophils. Data is mean ± s.e.m and statistical analyses was performed using Ordinary One-Way ANOVA, p=0.29 (overall for basal respiration), p=0.67 (overall for ATP production), p=0.38 (overall for maximal respiration). **(e)** Quantification of OCR in freshly isolated B-cells from aged (n=4, biological replicates), NR treated aged (n=3, biological replicates) and young (n=4, biological replicates) mice. **(f)** Basal respiration, ATP production and maximal respiration levels in aged, NR treated aged and young B-cells**.** Data is mean ± s.e.m and statistical analyses were performed using Ordinary One-Way ANOVA**,** p=0.30 (overall for basal respiration), p=0.16 (overall for ATP production), p=0.23 (overall for maximal respiration) **(g)** Quantification of percentages of CD69 positive macrophages, neutrophils and B-cells. Data is mean ± s.e.m and statistical analyses were performed using Ordinary One-way ANOVA, p=0.0036 (overall for macrophages), p=0.25 (overall for B-cells) or Kruskal-Wallis, p=0.28 (overall for neutrophils) with individual groups compared using the Tukey's multiple comparisons test for macrophages only. Abbreviations: NR=nicotinamide riboside, Y=young, Y+NR= NR treated young animal, A=aged, A+NR=NR treated aged animal, WBM=total bone marrow. Source data are provided as a Source Data File.

**Supplementary Fig. 7: NR administration does not significantly impact homing ability of aged HSC.**

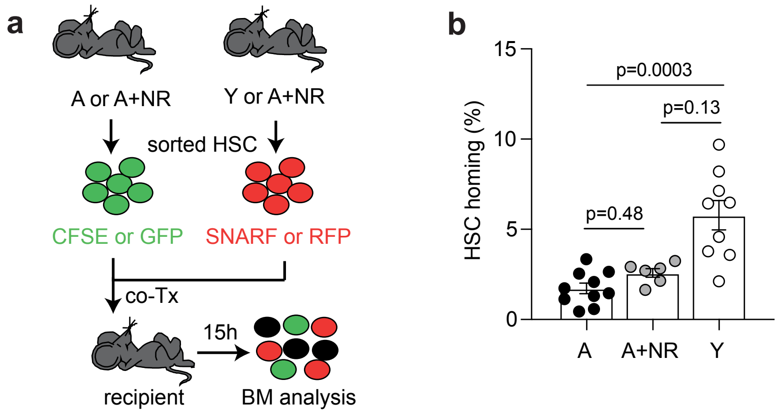

**Supplementary Fig. 7: NR administration does not significantly impact homing ability of aged HSC. (a)** Schematic representation of experimental set up for the analysis of HSC homing post-transplant. **(b)** Quantification of the frequency of aged or NR treated aged CFSE$^{+}$/GFP$^{+}$ (green) donor HSC (n=10, biological replicates), young SNARF$^{+}$/RFP$^{+}$ (red) donor HSC (n=9, biological replicates) or aged SNARF$^{+}$/RFP$^{+}$ (red) donor HSC from NR treated mice (n=6, biological replicates) homed to recipient BM 15 hours post-transplant. Data is mean ± s.e.m and is a pool from 3 separate experiments. Statistical analyses were performed using Kruskal-Wallis, p=0.0005 (overall) with individual groups compared using Dunn's multiple comparisons test, p-values indicated. Abbreviations: NR=nicotinamide riboside, A=aged, A+NR=NR treated aged animal, Y=Young, HSC=hematopoietic stem cells. Source data are provided as a Source Data File.

**Supplementary Fig. 8: Treatment of recipients with NR following the transplant of HSC from aged donors treated with NR does not significantly alter the size of the donor stem and progenitor pools.**

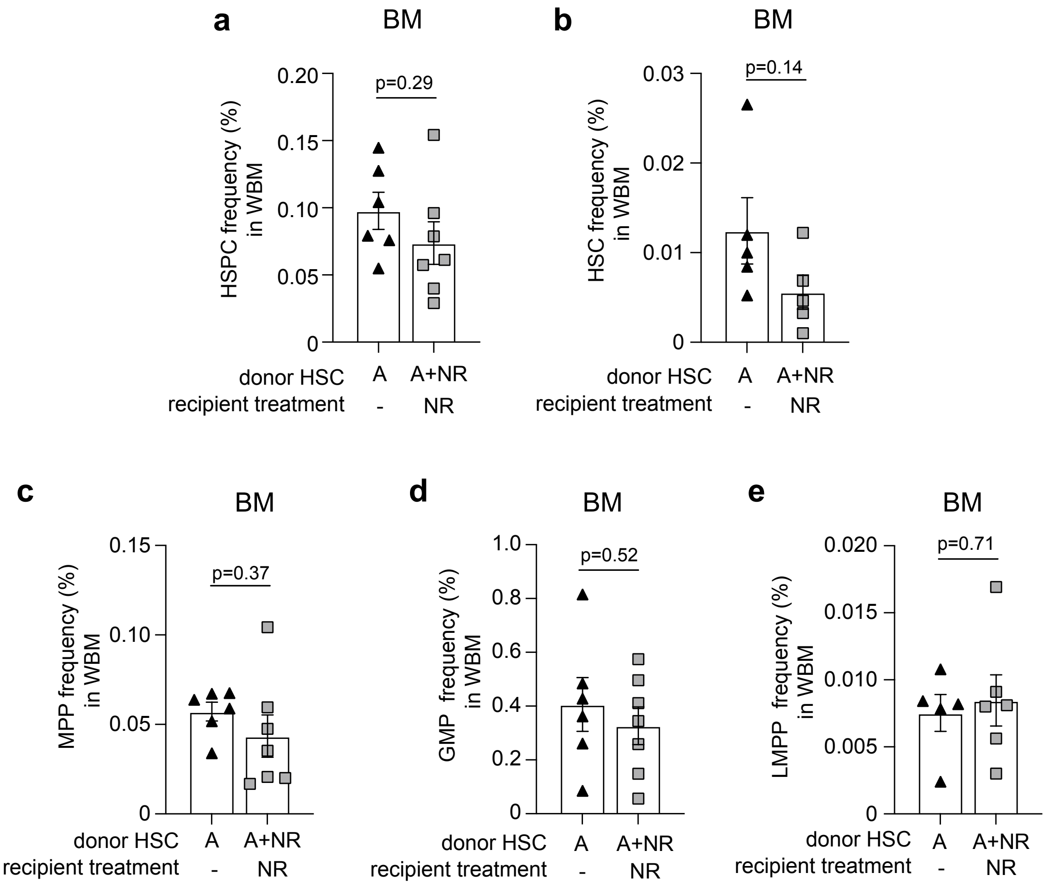

**Supplementary Fig. 8: Treatment of recipients with NR following the transplant of HSC from aged donors treated with NR does not significantly alter the size of the donor stem and progenitor pools**. Quantification of the frequency of donor-derived **(a)** HSPC **(b)** HSC **(c)** MPP **(d)** GMP and **(e)** LMPP cells in the BM of recipients 20 weeks following transplant of HSC from untreated or NR treated aged mice as described in Figure 6a. The frequency of each population is calculated as a proportion of $CD45^{+}$ cells derived from either untreated (n=6, biological replicates) or NR treated aged (n=7, biological replicates) HSC donors within each recipient. For recipients where <0.01% engraftment of the relevant cell population was observed, no lineage analysis was performed. Data is mean ± s.e.m and statistical analyses were performed using an unpaired two-sided Student's t-test, p-values indicated. Triangle symbols refer to untreated recipients and square symbols refer to NR treated recipients. Abbreviations: NR=nicotinamide riboside, A=aged, A+NR=NR treated aged animal, HSPC=hematopoietic stem and progenitor cells, HSC=hematopoietic stem cells, MMP=multipotent progenitors, GMP=granulocyte–macrophage progenitors, LMPP=Lymphoid-primed, MPP=Multi-potent progenitors, BM=Bone marrow, WBM=total bone marrow. Source data are provided as a Source Data File.

**Supplementary Fig. 9: NR treatment of recipients alone is insufficient for improved engraftment outcomes nor is *in vitro* pre-treatment of aged HSC.**

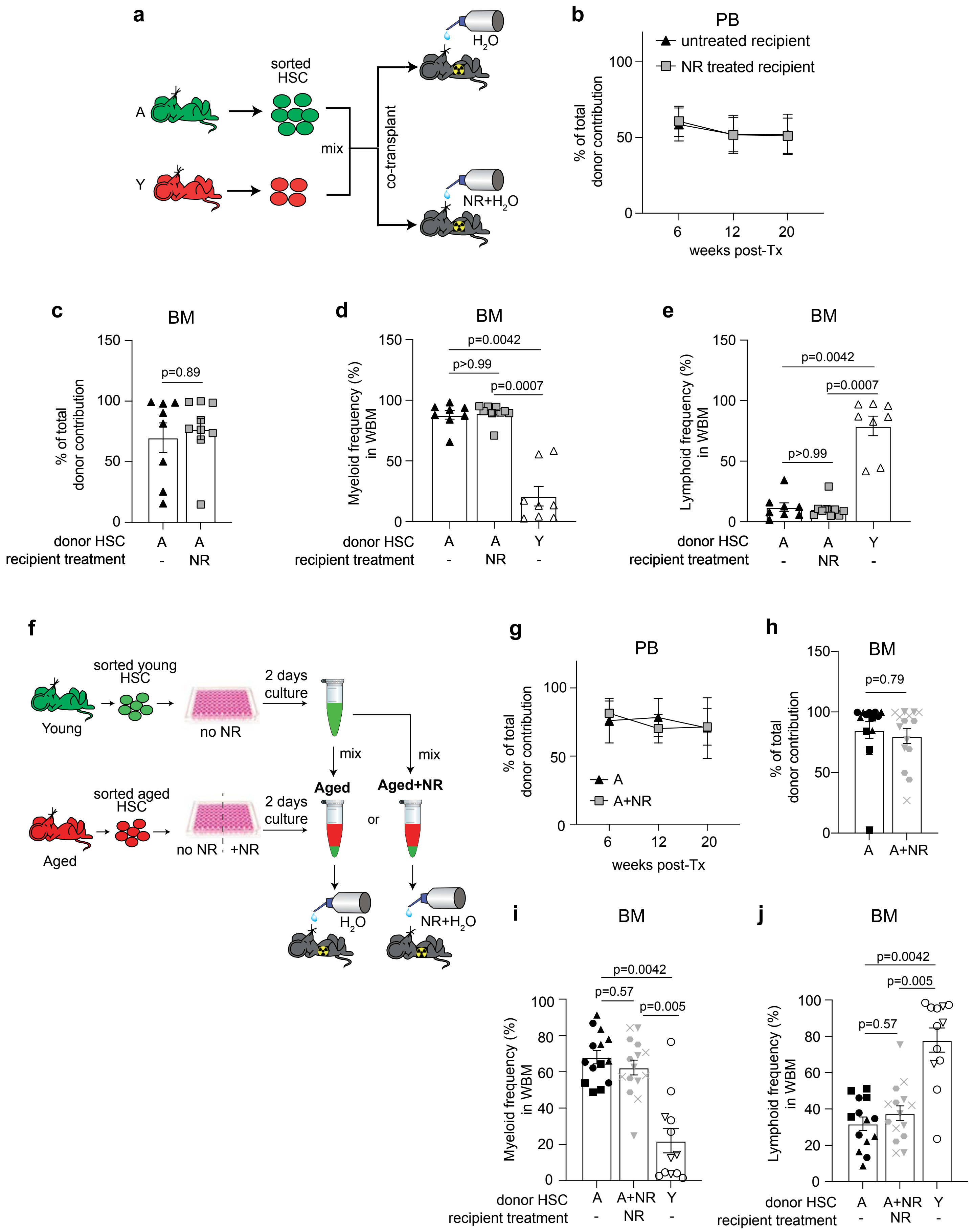

**Supplementary Fig. 9: NR treatment of recipients alone is insufficient for improved engraftment outcomes nor is *in vitro* pre-treatment of aged HSC. (a)** Schematic representation of the experimental set up for the competitive transplant experiment where aged GFP$^+$ HSC (n = 2 pooled) were co-transplanted with young RFP$^+$ HSC (n = 8 pooled) into untreated or NR treated recipient mice. **(b)** Quantification of the percentage of aged donor engraftment in untreated (n=8, biological recipients) and NR treated young recipient mice (n=9, biological recipients) relative to total donor derived hematopoietic cells in PB at 6, 12 and 20 weeks and **(c)** BM at 20 weeks following transplantation. Data is mean ± s.e.m and statistical analyses were performed using Two-Way ANOVA with Geisser-Greenhouse correction, p=0.98 (overall for treatment group) for **(b)** and two-sided Mann-Whitney U-test, p-value indicated for **(c)**. **(d, e)** Quantification of the frequency of aged HSC and young HSC (n=8; untreated recipients only) donor-derived **(d)** myeloid cells (Gr1/Mac-1$^+$) and **(e)** lymphoid cells (B220$^+$, CD3$^+$) in BM of recipient mice that were untreated (n=8, biological recipients) or treated with NR (n=9, biological recipients) 20 weeks after transplantation. Data is mean ± s.e.m and statistical analysis was performed using Kruskal-Wallis, p=0.0004 (overall for **d, e**) with individual groups compared using Dunn's multiple comparisons test, p-value indicated for **(d, e)**. **(f)** Schematic representation of the experimental set up for the competitive transplant experiment where aged tomato$^+$ donor cells were pre-treated with or without 1 mM NR *in vitro* followed by co-transplantation with young cultured GFP$^+$ HSC into untreated or NR treated mice, respectively. **(g)** Quantification of the percentage of aged (n=3, biological replicates averaged from 4 individual recipients) and NR treated aged (n=3, biological replicates averaged from 5 individual recipients) donor cells relative to total donor derived hematopoietic cells in PB of young recipient mice at 6, 12 and 20 weeks and **(h)** BM at 20 weeks following transplantation. For recipients where <0.01% engraftment of the relevant cell population was observed, no lineage analysis was performed. For **(g)**, data is

mean ± s.e.m. and each data point represents the average of all individual recipients and statistical analysis was performed on the biological replicates (n=3 for each of aged, NR treated aged and young) averaged from 4-5 individual biological recipients) using Two-Way ANOVA with Geisser-Greenhouse correction, p=0.95 (overall for treatment group). For **(h)**, data is mean ± s.e.m. and statistical analyses was performed using an unpaired two-sided Student's t-test, p-value indicated. Quantification of the frequency of aged and NR treated aged and young HSC donor-derived **(i)** myeloid cells (Gr1/Mac-$1^{+}$) and **(j)** lymphoid cells ($B220^{+}$, $CD3^{+}$) in BM of recipient mice 20 weeks after transplantation. Data is mean ± s.e.m and statistical analysis was performed using Ordinary One-Way ANOVA, p=0.0026 (overall) for **(i**, **j)**. Triangle symbols refer to untreated recipients and square symbols refer to NR treated recipients **(b-e, g)**. Each data point represents an individual recipient and the symbol shape defines the biological replicate group (3-5 recipients per biological replicate) **(h-j).** Abbreviations: NR=nicotinamide riboside, A=aged, Y=Young, HSPC=hematopoietic stem and progenitor cells, HSC=hematopoietic stem cells, PB=peripheral blood, BM=bone marrow, WBM=total bone marrow. Source data are provided as a Source Data File.

**Supplementary Fig. 10: NR treatment of aged HSC donors alone is insufficient for improved engraftment outcomes.**

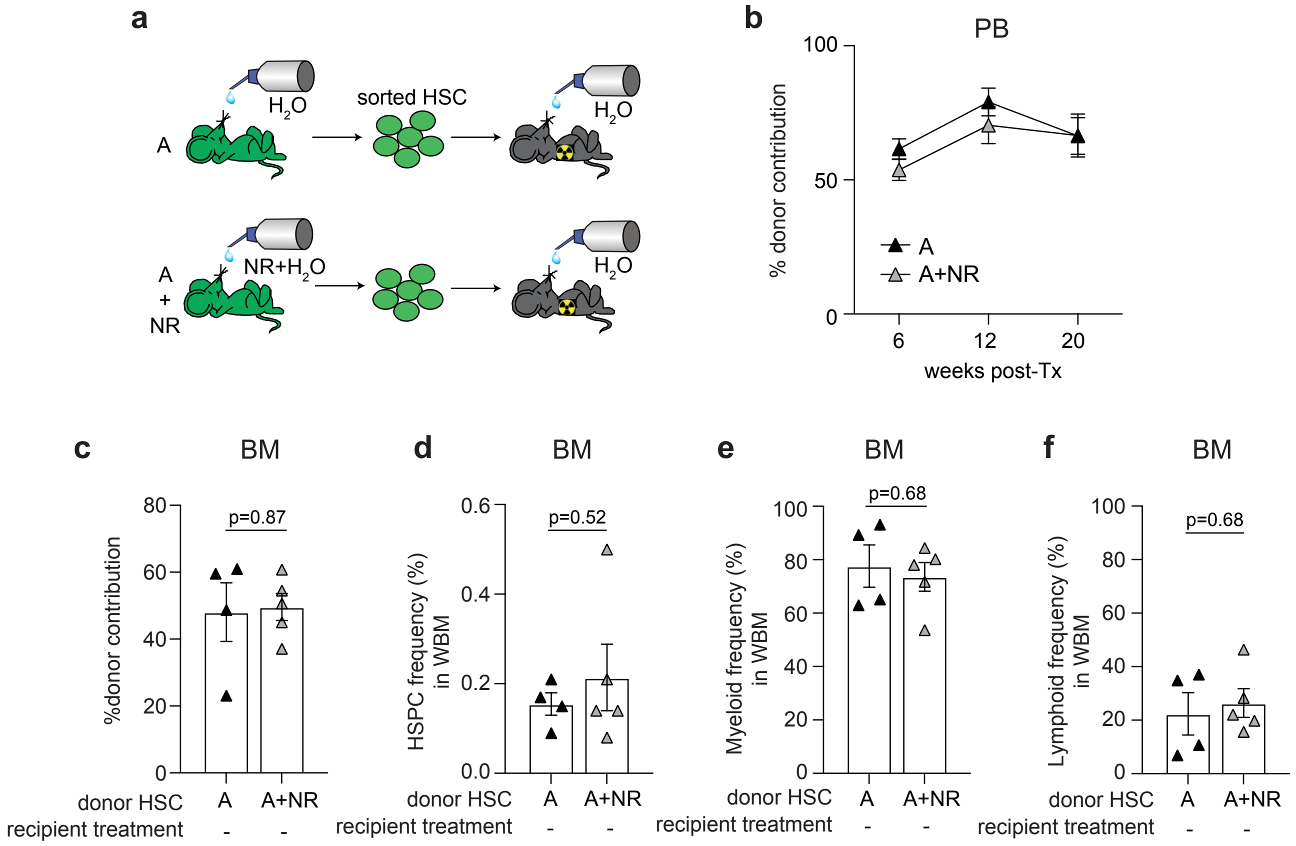

**Supplementary Fig. 10: NR treatment of aged HSC donors alone is insufficient for improved engraftment outcomes. (a)** Schematic representation of the experimental set up for the competitive transplant experiment where recipients were not treated with NR. **(b)** Quantification of the percentage of aged (n=4, biological replicates) and NR treated aged (n=5, biological replicates) $GFP^+$ donor cells relative to total donor derived hematopoietic cells in PB of young recipient mice at 6, 12 and 20 weeks and **(c)** BM at 20 weeks following transplantation. Quantification of the frequency of aged and NR treated aged HSC donor-derived **(d)** HSPC ($Lin^-Sca\text{-}1^+cKit^+$) **(e)** myeloid cells ($Gr1/Mac\text{-}1^+$) and **(f)** lymphoid cells ($B220^+$, $CD3^+$) in BM of recipient mice 20 weeks after transplantation. Data is mean ± s.e.m and statistical analyses were performed using Two-way ANOVA, p=0.52 (overall) for **(b)**, an unpaired two-sided Student's t-test, p-values indicated for **(c-f)**. Abbreviations: NR=nicotinamide riboside, A=aged, A+NR=NR treated aged animal, Y=Young, HSPC=hematopoietic stem and progenitor cells, HSC=hematopoietic stem cells, PB=peripheral blood, BM=bone marrow, WBM=total bone marrow. Source data are provided as a Source Data File.

## Supplementary Table 1: Anti-mouse antibodies used for flow cytometry

| Target Protein | Fluorophore | Clone | Isotype | Supplier | Cat # | Concentration |
|---|---|---|---|---|---|---|
| CD3e | BV510<br>BV785<br>APCCy7 | 17A2<br>17A2<br>17A2 | rat IgG2b<br>rat IgG2b<br>rat IgG2b | Biolegend<br>Biolegend<br>Biolegend | 100234<br>100231<br>100222 | 2 μg/ml<br>0.5 μg/ml<br>0.5 μg/ml |
| CD45R, B220 | APCCy7<br>BV510<br>BV785<br>AF647<br>purified | RA3_6B2<br>RA3_6B2<br>RA3_6B2<br>RA3_6B2<br>RA3_6B2 | rat IgG2a<br>rat IgG2a<br>rat IgG2a<br>rat IgG2a<br>rat IgG2a | BD<br>BD<br>Biolegend<br>Biolegend<br>BD | 552094<br>563103<br>103246<br>103226<br>557390 | 0.5 μg/ml<br>2 μg/ml<br>1 μg/ml<br>1 μg/ml<br>1 μg/ml |
| Gr1-, Ly6G | APCCy7<br>AF647<br>purified | RB6-8C5<br>RB6-8C5<br>RB6-8C5 | rat IgG2b<br>rat IgG2b<br>rat IgG2b | BD<br>Biolegend<br>BD | 557661<br>108418<br>553123 | 0.2 μg/ml<br>0.2 μg/ml<br>1 μg/ml |
| Mac-1, CD11b | APCCy7<br>AF647<br>FITC<br>purified | M1/70<br>M1/70<br>M1/70<br>M1/70 | rat IgG2b<br>rat IgG2b<br>rat IgG2b<br>rat IgG2b | BD<br>Biolegend<br>BD<br>BD | 557657<br>101218<br>553310<br>553308 | 0.5 μg/ml<br>0.2 μg/ml<br>1 μg/ml<br>1 μg/ml |
| CD117,<br>c-Kit | BUV395<br>AF647 | 2B8 | rat IgG2b | BD<br>Biolegend | 564011<br>105818 | 1 μg/ml<br>1 μg/ml |
| Sca-1, Ly-6A/E | PECy7 | E13-161.7 | rat IgG2a | Biolegend | 122514 | 0.4 μg/ml |
| TER119 | APCCy7<br>BV510<br>purified<br>PE | TER119<br>TER119<br>TER119<br>TER119 | rat IgG2b<br>rat IgG2b<br>rat IgG2b<br>rat IgG2b | BD<br>BD<br>BD<br>BD | 560509<br>563995<br>550565<br>553673 | 5 μg/ml<br>5 μg/ml<br>0.06 μg/ml<br>1 μg/ml |
| CD16/32 | BV711 | 2.4G2 | rat IgG2b | BD | 740659 | 2 μg/ml |
| CD34 | AF700 | RAM34 | rat IgG2a | BD | 560518 | 5 μg/ml |
| CD48 | APC<br>FITC<br>BV421<br>BV510<br>biotin | HM48-1<br>HM48-1<br>HM48-1<br>HM48-1<br>HM48-1 | A. Hamster<br>IgG1 | BD<br>BD<br>BD<br>BD<br>Biolegend | 562746<br>557484<br>562745<br>563536<br>103410 | 0.5 μg/ml<br>1 μg/ml<br>1 μg/ml<br>2 μg/m<br>1 μg/ml |
| CD127, IL17Rα | BV605 | A7R34 | rat IgG2a | Biolegend | 135041 | 5 μg/ml |
| CD135, Flt3 | Biotin | A2F10 | rat IgG2a | Biolegend | 135308 | 25 μg/ml |
| CD150<br>(SLAMF1) | BV650<br>PE | TC15-<br>12F12.2<br>TC15-<br>12F12.2 | rat IgG2a<br>rat IgG2a | Biolegend<br>Biolegend | 115931<br>115904 | 0.25 μg/ml<br>0.1 μg/ml |
| Ly6C | BV711 | HK1.4 | rat IgG2c | Biolegend | 128037 | 0.1 μg/ml |
| Ly6G | APCcy7<br>AF700 | 1A8<br>1A8 | rat IgG2a<br>rat IgG2a | BD<br>Biolegend | 560600<br>127621 | 0.4 μg/ml<br>1 μg/ml |
| F4/80 | AF647 | CI-A3-1 | Rat IgG2b | BD | NB600-<br>404 | 0.5 μg/ml |
| Ki-67 | BV786 | B56 | mouse IgG1 | BD | 563756 | 5 μl/test |
| CD69 | BV650 | H1.2F3 | A. Hamster IgG | Biolegend | 104541 | 2.5 μg/ml |
| CD11c | PE | N418 | A. Hamster IgG | Biolegend | 117308 | 0.25 μg/ml |
| Streptavidin | BUV805 | | | BD | 564923 | 1 μg/ml |
