# Supplementary material for: Nicotinamide riboside attenuates age-associated metabolic and functional changes in hematopoietic stem cells
Source: Nat Commun. 2021 May 11;12:2665. doi: 10.1038/s41467-021-22863-0 (PMC8113506; doi:10.1038/s41467-021-22863-0)
Supplement: Supplementary file 2 — Description of Additional Supplementary Files [file 41467_2021_22863_MOESM2_ESM.pdf]

Description of Additional Supplementary Files

File name: Supplementary Data 1
Description: Differentially expressed genes between young HSC compared to aged HSC.

File name: Supplementary Data 2
Description: Differentially expressed genes between young HSC compared to NR treated aged HSC.

File name: Supplementary Data 3
Description: Differentially expressed genes between aged HSC compared to NR treated aged HSC.
