# Supplementary material for: Nicotinamide riboside attenuates age-associated metabolic and functional changes in hematopoietic stem cells
Source: Nat Commun. 2021 May 11;12:2665. doi: 10.1038/s41467-021-22863-0 (PMC8113506; doi:10.1038/s41467-021-22863-0)
Supplement: Supplementary file 6 — Reporting Summary [file 41467_2021_22863_MOESM6_ESM.pdf]

nature research

Corresponding author(s): Susan Nilsson
Christian Nefzger

Last updated by author(s): Mar 12, 2021

Reporting Summary

Nature Research wishes to improve the reproducibility of the work that we publish. This form provides structure for consistency and transparency in reporting. For further information on Nature Research policies, see Authors & Referees and the Editorial Policy Checklist.

Statistics

For all statistical analyses, confirm that the following items are present in the figure legend, table legend, main text, or Methods section.

n/a	Confirmed	
☐	☒	The exact sample size (*n*) for each experimental group/condition, given as a discrete number and unit of measurement
☐	☒	A statement on whether measurements were taken from distinct samples or whether the same sample was measured repeatedly
☐	☒	The statistical test(s) used AND whether they are one- or two-sided<br>*Only common tests should be described solely by name; describe more complex techniques in the Methods section.*
☐	☒	A description of all covariates tested
☐	☒	A description of any assumptions or corrections, such as tests of normality and adjustment for multiple comparisons
☐	☒	A full description of the statistical parameters including central tendency (e.g. means) or other basic estimates (e.g. regression coefficient) AND variation (e.g. standard deviation) or associated estimates of uncertainty (e.g. confidence intervals)
☐	☒	For null hypothesis testing, the test statistic (e.g. F, t, r) with confidence intervals, effect sizes, degrees of freedom and P value noted<br>*Give P values as exact values whenever suitable.*
☒	☐	For Bayesian analysis, information on the choice of priors and Markov chain Monte Carlo settings
☒	☐	For hierarchical and complex designs, identification of the appropriate level for tests and full reporting of outcomes
☒	☐	Estimates of effect sizes (e.g. Cohen's d, Pearson's r), indicating how they were calculated

Our web collection on statistics for biologists contains articles on many of the points above.

Software and code

Policy information about availability of computer code

Data collection	BD FACS Sortware 1.2.0.142 was used for Flow Cytometric Cell Sorting<br>BD FACSDIVA Software 8.01 was used for Flow Cytometric Analysis<br>EnSpire Manager 4.13.3005.1482 (PerkinElmer) was used for colourmetric assays<br>Agilent Seahorse Wave Desktop Version 2.2.1.5 was used for the seahorse assays<br>Jess Simple WesternTM platform was used for automated western blotting
Data analysis	GraphPad Prism 8 (GraphPad software Inc, San Diego, USA) was used for statistical analysis<br>FlowJo v10 was used for Flow Cytometric analysis<br>STAR, EdgeR, STRING-DB, DEGUST, DAVID, plotPCA, hclust, Multiple Experiment Viewer and CYTOSCAPE were used for RNAseq analysis<br>Compass for Simple Western 5.0.1 was used for automated western blot analysis

For manuscripts utilizing custom algorithms or software that are central to the research but not yet described in published literature, software must be made available to editors/reviewers. We strongly encourage code deposition in a community repository (e.g. GitHub). See the Nature Research guidelines for submitting code & software for further information.

Data

Policy information about availability of data

All manuscripts must include a data availability statement. This statement should provide the following information, where applicable:

- Accession codes, unique identifiers, or web links for publicly available datasets
- A list of figures that have associated raw data
- A description of any restrictions on data availability

Raw data for the RNA sequencing experiment will be made available in a public repository and accession numbers provided in the manuscript. Further data that support the findings of this study are available from the corresponding author upon reasonable request.

Field-specific reporting

Please select the one below that is the best fit for your research. If you are not sure, read the appropriate sections before making your selection.

☒ Life sciences ☐ Behavioural & social sciences ☐ Ecological, evolutionary & environmental sciences

For a reference copy of the document with all sections, see nature.com/documents/nr-reporting-summary-flat.pdf

Life sciences study design

All studies must disclose on these points even when the disclosure is negative.

Sample size	Sample sizes were chosen to provide sufficient statistical power to detect differences.
Data exclusions	No data was excluded
Replication	Data was replicated using multiple n, as well as biological repeats. There were no situations of unreproducibility
Randomization	Mice were always randomized into treatment groups
Blinding	Investigators performing the initial analysis and data collection were blinded as to which treatment group a sample belonged to

Reporting for specific materials, systems and methods

We require information from authors about some types of materials, experimental systems and methods used in many studies. Here, indicate whether each material, system or method listed is relevant to your study. If you are not sure if a list item applies to your research, read the appropriate section before selecting a response.

Materials & experimental systems

n/a	Involved in the study	
☐	☒	Antibodies
☒	☐	Eukaryotic cell lines
☒	☐	Palaeontology
☐	☒	Animals and other organisms
☒	☐	Human research participants
☒	☐	Clinical data

Methods

n/a	Involved in the study	
☒	☐	ChIP-seq
☐	☒	Flow cytometry
☒	☐	MRI-based neuroimaging

Antibodies

Antibodies used	The Supplier, catalogue number, clone, fluorophore and concentration of all antibodies used in the study are described in Supplementary Table 1
Validation	All antibodies were validated before use and example profile images have been included throughout the manuscript

Animals and other organisms

Policy information about studies involving animals; ARRIVE guidelines recommended for reporting animal research

Laboratory animals	The species, strain,sex and age of animals used are described in the methods section as well as in the results/figure legends where relevant
Wild animals	This study did not involve wild animals
Field-collected samples	This study did not involve samples collected from the field
Ethics oversight	Ethics oversight was done by the Monash Animal Ethics Committee (MARP) 3 & the University of Queensland Animal Ethics commitee

Note that full information on the approval of the study protocol must also be provided in the manuscript.

Flow Cytometry

Plots

Confirm that:

☒ The axis labels state the marker and fluorochrome used (e.g. CD4-FITC).

☒ The axis scales are clearly visible. Include numbers along axes only for bottom left plot of group (a 'group' is an analysis of identical markers).

☒ All plots are contour plots with outliers or pseudocolor plots.

☒ A numerical value for number of cells or percentage (with statistics) is provided.

Methodology

Sample preparation	Sample preparation is clearly described in the methods section
Instrument	The instruments used are clearly described in the methods section
Software	BD FACS Sortware 1.2.0.142 was used for Flow Cytometric Cell Sorting<br>BD FACSDIVA Software 8.01 was used for Flow Cytometric Analysis<br>FlowJo v10 was used for Flow Cytometric analysis
Cell population abundance	The abundance of populations and purities are described throughout the text
Gating strategy	Gating strategies are included in Supplemental Data

☒ Tick this box to confirm that a figure exemplifying the gating strategy is provided in the Supplementary Information.
